# High‐Resolution Insights Into Genetic Diversity and Population Structure of *Raja clavata* Across the Northeast Atlantic and Mediterranean Sea

**DOI:** 10.1002/ece3.74528

**Published:** 2026-11-13

**Authors:** Timo Michael Staeudle, Eleanor S. I. Greenway, Jurgen Batsleer, Cristina Rodríguez‐Cabello, Diana Catarino, Jim R. Ellis, Eva Giacomello, Pascal Lorance, Hendrik‐Jan Megens, Joey J. Portier, Nir Stern, Klaas Sys, Jan Jaap Poos

**Affiliations:** ^1^ Aquaculture Biology and Fisheries Ecology Group Wageningen University & Research Wageningen the Netherlands; ^2^ Wageningen Marine Research, Wageningen University & Research IJmuiden the Netherlands; ^3^ Instituto Español de Oceanografía Santander Spain; ^4^ Institute of Marine Sciences—OKEANOS, University of the Azores Horta Portugal; ^5^ Centre for Environment, Fisheries and Aquaculture Science Suffolk, Lowestoft UK; ^6^ DECOD (Ecosystem Dynamics and Sustainability), Institut Agro—Agrocampus Ouest, IFREMER, INRAE Nantes France; ^7^ Animal Breeding and Genomics Wageningen University and Research Wageningen the Netherlands; ^8^ Steinhardt Museum of Natural History, Tel Aviv University Tel Aviv Israel; ^9^ Animal Sciences Unit Flanders Research Institute for Agriculture, Fisheries and Food Oostende Belgium

**Keywords:** conservation, elasmobranch, genetic markers, isolation by distance, SNP

## Abstract

Understanding genetic population structures is crucial for stock assessment, fisheries management and the effective conservation of marine species. This is of particular relevance for elasmobranchs that are vulnerable to fishing pressure due to late maturation and low fecundity characteristics. *Raja clavata* , a commercially exploited skate species is one such elasmobranch. This study investigated the genetic diversity and population structure of *R. clavata* across the Northeast Atlantic and the Mediterranean Sea, on large (between ecoregions) and small (within ecoregions) geographic scales. Ecoregions covered are the Aegean‐Levantine Sea, Azores, Bay of Biscay and the Iberian Coast, Celtic Seas and Greater North Sea. A panel of 4793 single‐nucleotide polymorphisms was used across 6902 individuals, collected between 2001 and 2023, to assess genetic diversity and population structures of *R. clavata* in the Northeast Atlantic and the Mediterranean Sea. The analyses include principal component analyses, discriminant analysis of principal components, admixture, isolation by distance and genetic diversity indices. Evidence for at least five subpopulations is presented. Population differentiation is demonstrated between and within ecoregions, with clear genetic differentiation of a coastal subpopulation in the Bay of Biscay, revealing genetic structure on a relatively small geographic scale for elasmobranchs. Significant isolation by distance patterns were observed in the Greater North Sea and the Bay of Biscay and Iberian Coast ecoregions, while genetic diversity estimates differed substantially between ecoregions, with lower levels observed around the Azores, suggesting potential past genetic bottlenecks. This study highlights the importance of utilising updated genetic approaches to inform conservation and fisheries management strategies. Its sampling effort places it among the largest of its kind for an elasmobranch species and provides unprecedented high resolution genetic insights into the population structure of *R. clavata* .

## Graphical abstract

This study investigated the genetic diversity and population structure of *Raja clavata* across the Norteast Atlantic and the Mediterranean Sea, on large (between ecoregions) and small (within ecoregions) geographic scales. A panel of 4793 single nucleotide polymorphisms was used across 6902 individuals, collected between 2001 and 2023, to assess genetic diversity and population structures. Population differentiation is demonstrated between and within ecoregions, with clear genetic differentiation of a coastal subpopulation in the Bay of Biscay, revealing genetic structures on a relatively small geographic scale for elasmobranchs.

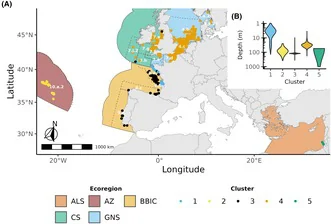

## 1 Introduction

Pressures from fishing activity have an impact on the size and structure of fish populations, their habitats and their life‐history traits (Delaval et al. 2022; Enberg et al. 2009; Jennings and Kaiser 1998; Stevens et al. 2000). Elasmobranchs, with their characteristically late maturation and low fecundity, are therefore especially vulnerable to fishing (Dulvy et al. 2014; Elliott et al. 2020), and with the industrialisation of bottom fishing, populations of several demersal elasmobranchs in European waters declined (Peristeraki et al. 2020; Sguotti et al. 2016). While examples exist of successful management efforts to reverse the declines for elasmobranchs (Amelot et al. 2021; Staeudle et al. 2024; Ward‐Paige et al. 2012), challenges for their sustainable management and conservation remain (Batsleer et al. 2024; Booth et al. 2019; Dedman et al. 2024).

Addressing the management of elasmobranch populations raises pertinent questions regarding the delineation of appropriate population boundaries (Reiss et al. 2009). This is crucial to the understanding of their population dynamics, including stock assessments, and implementing effective management strategies. Declines in elasmobranch populations are of concern because they become increasingly more susceptible to the consequences of small population dynamics, such as inbreeding and genetic drift (Domingues et al. 2018). These phenomena exacerbate the loss of genetic diversity within elasmobranch populations, which may act as a significant precursor to increased extinction risk (Frankham 2005; Lynch 1996; Nykänen et al. 2020). The diminished genetic diversity not only compromises the evolutionary potential of these species but also renders them more vulnerable to environmental perturbations and disease outbreaks (Frankham et al. 2002). Preserving genetic diversity of populations is thus a key priority for conservation (Lynch 1996; Nykänen et al. 2020).

Over the last two decades, genetic studies have improved our understanding of the genetic diversity of skates and rays, improving species identification, phylogeny and phylogeography (Crobe et al. 2021; Delaval et al. 2022; Ferrari et al. 2018; Serra‐Pereira et al. 2010). Genetic diversity between populations is shaped by mechanisms of mutation, selection, genetic drift and gene flow (Hallerman 2003; Marandel et al. 2018; Stearns and Hoekstra 2005). Among these mechanisms, gene flow, the transfer of genetic material between populations, is of high relevance for marine populations. In the marine environment, this occurs mostly through passive dispersal of gametes or larvae, or active dispersal of juvenile and adult individuals. Gene flow is generally high in marine populations and population differentiation is typically weak (Nielsen et al. 2009; Waples 1998). While some geographic features, such as ocean current patterns and sea floor topology, may restrict dispersal and thus promote population differentiation, most seas and oceans lack obvious barriers to dispersal (Waples 1998). Consequently, the pelagic larval stages of many marine species facilitate dispersal over large distances (Cowen and Sponaugle 2009). Active dispersal through long distance migration of adult marine organisms (Pittman and McAlpine 2003) may further hinder population differentiation (Frisk et al. 2014). Elasmobranchs, however, are oviparous or viviparous in the wild (Awruch 2015; Compagno 1990), with rare cases of parthenogenesis (Awruch 2015; Esposito et al. 2024; Feldheim et al. 2023). The main dispersal mechanism for their populations is therefore linked to the movements of adults (Grubbs 2010). Movements and annual migrations of adults have been observed for many skate species (Rajiformes; Bird et al. 2020; Hunter et al. 2005), a taxonomic group that is facing overexploitation through targeted fisheries and bycatch (Dulvy et al. 2014; Figueiredo et al. 2020).

*Raja clavata* (Linnaeus, 1758), also known as thornback ray or thornback skate, is commercially exploited throughout its distribution range (Figueiredo et al. 2020; International Council for the Exploration of the Sea [ICES] 2023; Walker and Hislop 1998). It is commonly found in the Northeast Atlantic, ranging from Iceland and Norway to the (ICES 2023) Azores, Madeira, Canary Islands and Morocco, as well as the Mediterranean Sea and Baltic Sea (Stehmann and Bürkel 1984). It inhabits a broad range of benthic habitats, from shallow coastal waters to depths exceeding 600 m, across a variety of sediment types (Heessen et al. 2015; Mytilineou et al. 2005; Santos et al. 2021). This skate species is known to prey on a variety of benthic and semi‐pelagic organisms such as crabs, shrimps, molluscs and small fish (Greenway et al. 2025; Gül and Demirel 2022; Šantić et al. 2012). *R. clavata* is oviparous, with the rectangular egg cases deposited on the seabed, where the incubation period is temperature dependent, lasting between 4 and 5.5 months (Clark 1922; Ellis and Shackley 1995). Tagging studies have suggested repeated seasonal migrations and spawning site fidelity (Hunter et al. 2005), which may limit mixing between local populations.

Previous studies on the population genetics of *R. clavata* in the Northeast Atlantic, and the Mediterranean Sea, found strong regional differentiation between the Mediterranean basin, the European continental shelf (Chevolot et al. 2006; Pasolini et al. 2011) and the Azores (Ball et al. 2016; Chevolot et al. 2006). There is also evidence of genetically structured populations within the Mediterranean Sea (Melis et al. 2023), and evidence for reduced genetic diversity in the Balearic Islands (western Mediterranean) due to past exploitation (Ferragut‐Perello et al. 2023). These studies used molecular markers found within the mitochondrial DNA (mtDNA), except for one study using nuclear microsatellites (Chevolot et al. 2006). Advances in next generation sequencing (NGS) technologies allow cost‐effective and reliable sequencing of thousands of single‐nucleotide polymorphisms (SNPs) across species' genomes. Large numbers of SNP markers have the potential to contribute to more precise and accurate parameter estimations and inferences for conservation (Allendorf et al. 2010), leading to improved insights into the delineation of fisheries stocks or natural marine populations (Cuéllar‐Pinzón et al. 2016; Spies et al. 2021; Waples et al. 2008; Zimmerman et al. 2020). At the same time, NGS technologies can provide molecular markers across both coding and non‐coding regions of the genome, allowing for investigations into fish genomics (Diopere et al. 2013), linking SNPs to bioclimatic variables in a large coastal shark (Klein et al. 2024) or revealing gene‐associated markers in an apex predatory shark (Domingues et al. 2022).

A recent study developed a panel of SNP markers for *R. clavata* , targeting several thousand SNPs. It was developed by demultiplexed sequences of *R. clavata* aligned to the reference genome of *Leucoraja erinacea* . Genotypes were scored via a clustering algorithm against 7726 samples, including duplicates, from mostly the Bay of Biscay, but also the Mediterranean Sea and western Iberia (Le Cam et al. 2019). This panel was subsequently used for the detection of closely related individuals and provided evidence for the existence of two distinct local populations of *R. clavata* in the Bay of Biscay, with limited demographic connectivity (Trenkel et al. 2022). These SNPs have been further utilised to identify and interpret sex‐linked SNP markers for sex assignment of *R. clavata* specimens (Trenkel et al. 2020).

This study applied this recently developed SNP panel for *R. clavata* to investigate patterns of population genetic structure in this commercially important elasmobranch species across five ecoregions in the Northeast Atlantic and Mediterranean Sea, on large (between ecoregions) and small (within ecoregions) spatial scales. The study aimed to examine population structure, using several thousand individuals, and to explore the genetic diversity, informing stock delineation efforts and conservation management.

## 2 Methods

### 2.1 Sample Collection

Tissue samples of *R. clavata* were collected by scientific observers on board research vessels and commercial fishing vessels, including a sub‐sample that had been supplied by the University of Groningen and used in a previous study (Chevolot et al. 2006). Additional fin clips were also sampled from commercially landed individuals at fish auctions in The Netherlands. Total length (cm) and the sex of each sampled individual were recorded, and the geographic location of samples was recorded as either the latitude and longitude of sampled hauls or as ecoregion when an exact geographic location was not available.

A total of 9144 tissue samples identified at collection as *R. clavata* were genotyped. Samples were collected from 2001 to 2023 from five ICES ecoregions: the Aegean‐Levantine Sea (ALS, *n* = 20), Azores (AZ, *n* = 108), Bay of Biscay and Iberian Coast (BBIC; *n* = 4118), Celtic Seas (CS, *n* = 299) and Greater North Sea (GNS, *n* = 4599) (Table 1; Figure 1). Most of the sampling in the GNS ecoregion occurred between 2017 and 2023, when tissue samples were collected at fish auctions, landing ports or during scientific research vessel cruises as part of the International Bottom Trawl Survey (IBTS) and Beam Trawl Survey (BTS). Additional samples from the CS and GNS ecoregions were obtained from a tissue sample collection held at the University of Groningen. These samples were collected in 2003–2004 and analysed in earlier studies (e.g., Chevolot et al. 2006). Samples from the BBIC ecoregion were provided from earlier studies (e.g., Trenkel et al. 2020, 2022). These samples were initially collected from fish auctions at landing ports, on board commercial fishing vessels and during scientific research vessel surveys, from 2011 to 2022. Samples from AZ were collected during scientific research vessel cruises (ARQDAÇO longline surveys) from 2001 to 2023, and samples from the ALS ecoregion were collected along the southern coast of Israel in 2022. An additional seven tissue samples of *R. brachyura* and two samples of *R. montagui* , collected in the GNS ecoregion, were genotyped and used as control samples to detect misidentifications of *R. clavata* in the analyses, because these species are sympatric with *R. clavata* across much of its geographic range.

**TABLE 1:** Overview of sample sizes of *Raja clavata* in the five ecoregions (Start), after removal of misidentified and intentional replicates, and sample sizes used for genomic analyses after filtering. Sample sizes after filtering are also given by sex (F = females, M = males and U = unknown sex).

Ecoregion	Period	Sample size			Post‐filtering by sex		
		Start	Misidentified and intentional replicated samples removed	Post‐filtering	F	M	U
ALS	2022–2022	20	20	19	0	0	19
AZ	2001–2023	108	103	97	52	43	2
BBIC	2011–2022	4118	3914	3069	1579	1490	0
CS	2003–2004	299	298	177	82	95	0
GNS	2003–2023	4599	4516	3540	1972	1557	11
Total	2001–2023	9144	8851	6902	3685	3182	32

Abbreviations: ALS = Aegean‐Levantine Sea, AZ = Azores, BBIC = Bay of Biscay and the Iberian Coast, CS = Celtic Seas, GNS = Greater North Sea.

**FIGURE 1:**
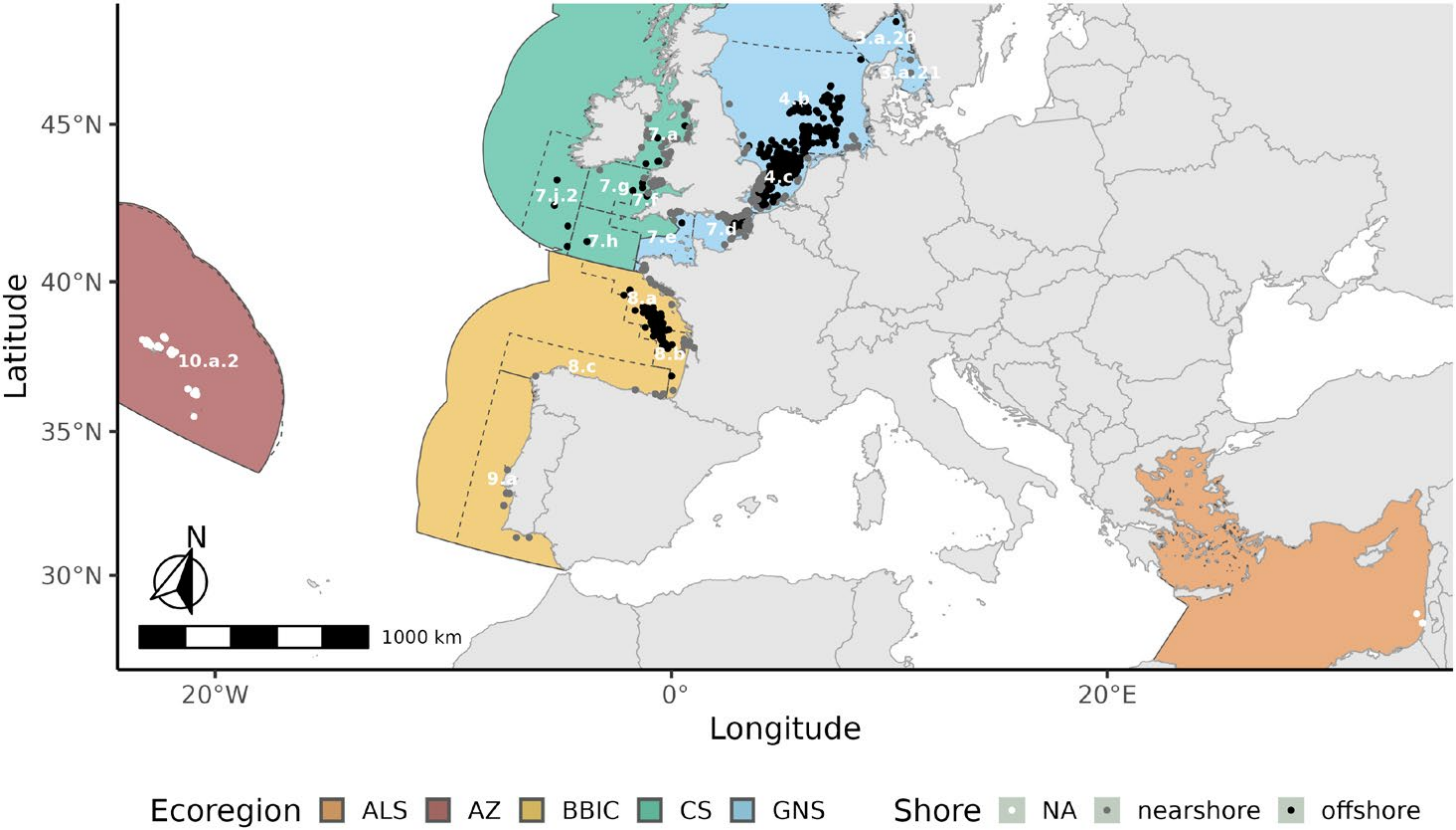
Sample locations of *Raja clavata* across the Northeast Atlantic Ocean and Mediterranean Sea are represented by dots. Grey dots indicate nearshore samples (< 30 km from the coast), black dots indicate offshore samples (≥ 30 km from coast) and white dots representing samples from the Azores and the Mediterranean Sea, where distance to shore classification was irrelevant for this study. Ecoregions are indicated with different colours, and relevant ICES divisions are indicated by white text, and delineated by dark grey dashed lines. For abbreviations, see Table 1.

### 2.2 Sample Genotyping and Filtering

All individuals were genotyped using an InfiniumXT iSelect‐96 SNP‐array with 9120 SNPs (Le Cam et al. 2019). Genotyping was performed by Labogena, where an initial quality‐control procedure resulted in the selection of 7042 SNPs for genotyping across all samples. To ensure that only reliably genotyped individuals and SNPs were used for analyses, several filtering steps were subsequently applied (Table 2).

**TABLE 2:** Summary of filtering steps for genotyped samples and single‐nucleotide polymorphism (SNP) data for *Raja clavata* . Samples were collected across the Northeast Atlantic and Mediterranean Sea.

Filtering step	#Samples	#SNP
Start	9144	9120
Misidentified RJC samples removed	9112	9120
Remove intentional replicates, non‐scoring loci and monomorphic loci	8851	7042
Sample call rate ≥ 0.95	6940	5977
Loci call rate ≥ 0.95	6940	5738
MAF threshold < 0.05	6940	5641
Remove unintentional replicates	6931	5641
Remove sex‐linked loci	6931	5619
Linkage disequilibrium > 0.2	6931	4793
Remove loci not in Hardy–Weinberg equilibrium for kin detection	6931	4294
Remove 1 sample from detected kin pairs (PO, FS, HS)	6902	4793

*Note:* #Samples represents the number of samples genotyped and #SNP represents the number of SNPs after each filtering step. Kin pairs identified included parent‐offspring (PO), full‐siblings (FS) and half‐siblings (HS).

First, correct species identification of *R. clavata* was assessed by comparing genotypes from all samples with those of *R. brachyura* and *R. montagui* control samples. The control samples from these two species consistently had high SNP call rates (> 0.8) and low heterozygosity rates (< 200 SNPs), compared with most other samples (Figure A1). In total, 32 samples initially identified as *R. clavata* clustered with the congener controls and were therefore considered likely misidentified and removed from further analyses.

Intentional sample replicates were removed, but used for genotyping error estimation with *Tiger* (Bresadola et al. 2020), required for two subsequent filtering steps: unintentional replicate detection and identifying kinship pairs. The set of replicates consisted of 132 duplicates and 49 triplicates.

Non‐scoring and monomorphic SNPs were removed after each sample filtering step using the ‘dartR’ package (V2.7.2; Gruber et al. 2018) in *R* (v4.2.3; R Core Team 2023). Samples and loci were filtered using a minimum call‐rate threshold ≥ 0.95 to ensure only reliably genotyped samples were retained for subsequent filtering steps and analyses. SNPs with minor allele frequencies (MAFs) < 0.05 in at least three ecoregions were subsequently removed using the ‘dartR’ package.

In addition to the intentional sample replicates, unintentional replicates could have been introduced by human error during sampling. Unintentional replicates were therefore identified as sample pairs with ≤ 1% mismatching loci using the R package ‘CKMRsim’ (v0.1.2.999; Anderson 2022). One sample from each pair was subsequently removed.

Sex‐linked SNP markers were then removed. Sex linkage of SNPs was assessed based on heterozygote excess (> 5×) in one sex across all samples and by comparison with previously identified sex‐linked SNPs on the panel. This led to the removal of 22 SNPs from the SNP panel (Table 2; Table A1).

To account for linkage disequilibrium, SNPs with the lower call rate were removed from SNP pairs found to be in pairwise linkage disequilibrium (*R* ^2^ > 0.2) using ‘dartR’. This reduces collinearity among markers while maximising SNP retention for downstream population‐structure analyses. This also ensured that no pairs of loci remained that had been identified as being within 50 nucleotides of each other during SNP‐array development.

To minimise potential bias from related individuals in population genomic analyses, first‐ and second‐order kinship relationships were identified using the R package ‘CKMRsim’. Prior to kinship inference, SNPs were filtered using an ecoregion‐level ‘Out Any’ Hardy–Weinberg equilibrium (HWE) approach (Pearman et al. 2022). A Benjamini and Hochberg (1995) correction for multiple testing was applied to control the false discovery rate. Kinship pairs within ecoregions were then identified using the ‘CKMRsim’ package. Once kinship pairs were identified, one individual from each related pair was removed from the dataset.

### 2.3 Population Structure

Spatial population structure was assessed using three approaches: principal component analyses (PCA), discriminant analyses and admixture modelling. Genetic clustering was evaluated by PCA, a model‐free ordination technique, and by discriminant analysis of principal components (DAPC) with both prior and de‐novo grouping. Across PCA and DAPC analyses, missing data were replaced by mean allele frequencies across all samples to minimise any potential bias. Ancestry proportions were estimated using two complementary methods for cross‐validation: the sparse Non‐Negative Matrix Factorisation (sNMF) algorithm, implemented in LEA, and the ADMIXTURE program. Both admixture analyses natively handle missing data (Alexander et al. 2009; Frichot and François 2015; Frichot et al. 2014).

### 2.4 Principal Component Analysis

Initial genetic structure was assessed using PCA, a model‐free ordination method, implemented with the R package ‘adegenet’ (v2.1.10; Jombart 2008; Jombart and Ahmed 2011).

### 2.5 DAPC With Prior Grouping

Spatial population structure was assessed using a DAPC with prior population grouping (Jombart et al. 2010) using the R package ‘adegenet’. Missing genotypes were replaced by mean allele frequencies across all samples to reduce bias. DAPCs with prior geographic population groupings of individuals were performed across two nested spatial scales.

Samples were either grouped by (i) ecoregion or (ii) classified into a nested set using a combination of ICES Division and coastal zone classification for the GNS, CS and BBIC ecoregions (‘ICES Division and coastal zone’ from here on). This coastal zone classification distinguished nearshore (< 30 km) and offshore (≥ 30 km) areas, relative to the coastlines of the United Kingdom (UK), Ireland (IRL) and continental Europe (CE). Samples from AZ were further assigned to the nearest island from the sampling location. Samples from the ALS were aggregated into a single group, given the small sample size and close proximity of sampling locations. Only ecoregion subsets with ≥ 15 samples were retained for analysis, exceeding the minimum sample size of 8–10 recommended for other organisms (Li et al. 2020; Nazareno et al. 2017; Scaketti et al. 2025). A summary of the sample sizes by ICES Division and coastal zone classification areas is provided in Appendix A (Table A2).

DAPC relies on dimension reduction of data using PCA. The number of retained principal components (PCs) for the DAPC was determined using cross‐validation. The training set for cross‐validation comprised 90% of the observations in each prior population group. DAPC results from the training set were then used to predict group membership for the remaining 10% of observations. This procedure was repeated 200 times for 5–400 PCA axes, in increments of five. The number of PCA axes associated with the lowest mean squared error was retained in the DAPC.

### 2.6 DAPC De Novo Clusters

A DAPC was performed using de novo grouping of individuals based on the number of clusters (*K*) identified with the *find.clusters*() function from ‘adegenet’, after replacing missing data with mean allele frequencies across all samples. This procedure first transformed genotype data using a PCA, followed by successive *k*‐means clustering with an increasing number of clusters. The maximum number of clusters evaluated was 10, and the optimal number of clusters was evaluated using the Bayesian Information Criterion (BIC) and the difference in BIC between subsequent clusters. For all DAPCs, the optimal number of PCs to use in the DAPC was determined using cross‐validation with a 0.9 training set, among the retained PC axes.

### 2.7 sNMF Ancestral Analysis

An admixture analysis was conducted using sNMF, implemented in the snmf() function in the R package ‘LEA’ (v3.10.2; Frichot and François 2015). sNMF estimates individual admixture coefficients from the genotypic matrix that represent proportions of individual genomes that originate from multiple ancestral gene pools (Frichot et al. 2014). It produces results comparable to those of Bayesian clustering programs such as STRUCTURE (Francois and Durand 2010; Pritchard et al. 2000). It assumes *K* ancestral populations and provides least‐squares estimates of ancestry proportions rather than maximum likelihood estimates (Frichot et al. 2014). Ten repetitions for each value of *K* (ranging from 1 to 10) were performed, and cross‐entropy criteria were calculated to assess the most likely value of *K*. Missing data are accounted for natively by the sNMF algorithm.

### 2.8 ADMIXTURE Ancestral Analysis

For cross‐verification purposes of ancestry proportions, the ADMIXTURE program (Alexander et al. 2009) was run for *K* values ranging from 1 to 10, with 10 repetitions and 10 cross‐validations for each *K*. Output for each value of *K* was aligned and merged using the R package ‘pophelper’ (v2.3.1; Francis 2017) to account for multimodality across runs.

### 2.9 Depth Analysis

Depth distributions were compared among the de novo clusters by matching sample locations, from all but the AZ ecoregion, with bathymetry data from the National Oceanic and Atmospheric Administration (NOAA), using the ‘marmap’ R package (v.1.0.10; Pante and Simon‐Bouhet 2013). Since sample depth in the AZ ecoregion was recorded at gear deployment, and 50 m depth strata were defined along the fishing set, each sample was assigned to the midpoint of its respective stratum. Pairwise comparisons of sample depths between ecoregions were tested with Dunn's test, using Holm's method (Holm 1979) to adjust *p*‐values for multiple comparisons.

### 2.10 Population Fixation Indices

Population differentiation was further assessed using Wright's fixation index (*F*_ST_; Wright 1949). Pairwise *F*_ST_ values were calculated for all pairwise combinations of areas containing ≥ 15 samples, using the Weir and Cockerham (1984) estimator adjusted for differing population sizes. These calculations were performed using the ‘StAMPP’ package (v.1.6.3; Pembleton et al. 2013) in R. Significance was tested against 1000 permutations, applying Holm's method to control the family‐wise error rate (Holm 1979).

To assess the relationship between genetic differentiation and geographical distance, matrix correlations were calculated between linearised pairwise *F*_ST_ estimates (Rousset 1997) and log‐transformed geographical distances between samples using the *mantel()* function in the ‘vegan’ R package (v.2.6‐8; Oksanen et al. 2024). Geographical distances were estimated as the least‐cost distances between pairs of locations with a minimum water depth of 0.1 m. These least‐cost distances were calculated using the *trans.mat()* function in the ‘marmap’ R package. The significance of the Mantel correlation statistic was evaluated using 10,000 permutations of rows and columns of the linearised pairwise *F*_ST_ estimates. These calculations were performed for the entire dataset, among the GNS, CS and BBIC ecoregion subsets, and for data within ecoregions, if sample depth allowed.

### 2.11 Genetic Diversity

Genetic diversity estimates, including observed heterozygosity (*H*_o_), expected heterozygosity (*H*_s_), inbreeding coefficient (*F*_IS_) and allelic richness (Ar), were calculated for each group based on ICES Division combined with coastal zone classifications, ecoregion and the suggested clusters by the DAPC with de novo grouping, using R package ‘hierfstat’ (v0.5‐11; Goudet 2005). Differences in genetic diversity estimates among groups were assessed using Dunn's test, with Holm's method (Holm 1979) used to adjust *p*‐values for multiple comparisons.

## 3 Results

### 3.1 SNP Statistics

Genotyping error rate of replicates was estimated at 0.003 for the unintentional replicate and kinship detection filtering steps. After filtering, 6902 genotyped samples and 4793 SNPs remained for subsequent analyses (Tables 1 and 2). The final set of SNPs had a mean call rate of 0.995 and an overall missing data rate of 0.0054. Mean heterozygosity was 0.338 (range 0.025–0.633), and mean MAF was 0.253 (range 0.049–0.500; Figure A2). Across ecoregions, mean SNP call rates were: 0.990 for ALS, 0.998 for AZ, 0.996 for BBIC, 0.992 for CS and 0.993 for GNS.

### 3.2 Principal Component Analysis

For the PCA, the first PC explained 0.021, the second PC explained 0.004 and the third PC explained 0.002 of the variance detected among the genotyped samples (Figure A3A). Visualising PCs 1–3 revealed separation of samples into three distinct clusters that broadly corresponded to ecoregions. Samples from the ALS and the Azores each formed two distinct clusters, whereas samples from the GNS, CS and BBIC formed a third cluster with partial overlap among these ecoregions (Figure A3B,C). Samples collected in the BBIC ecoregion clustered mostly separately from samples in the GNS and CS along PC1 (Figure A3B), with a notable exception of samples collected from 7.e_nearshore_CE. These samples clustered mostly with samples from the BBIC, while some overlapped with samples from GNS and CS. A notable separation of clusters of samples was observed for samples from the BBIC along PC3 (Figure A3C). Samples collected from the ICES divisions 8.a and 8.b clustered closely when collected offshore (8.a_offshore and 8.b_offshore). In contrast, samples from nearshore areas (8.a_nearshore and 8.b_nearshore) formed more distinct clusters, both from each other and from their offshore counterparts.

### 3.3 DAPC With Ecoregion Prior Grouping

Cross‐validation for the DAPC with ecoregion as prior grouping suggested retaining 290 PC axes (Figure 2B), with a 0.955 mean successful assignment and a root mean squared error of 0.048. The variances explained by the DAPC's linear discriminants (LD) are shown in Figure 2C. Three clusters were observed: one cluster comprising samples from the AZ, a second comprising samples from ALS and a third comprising partially overlapping sub‐clusters of the GNS, CS and BBIC ecoregions (Figure 2A).

**FIGURE 2:**
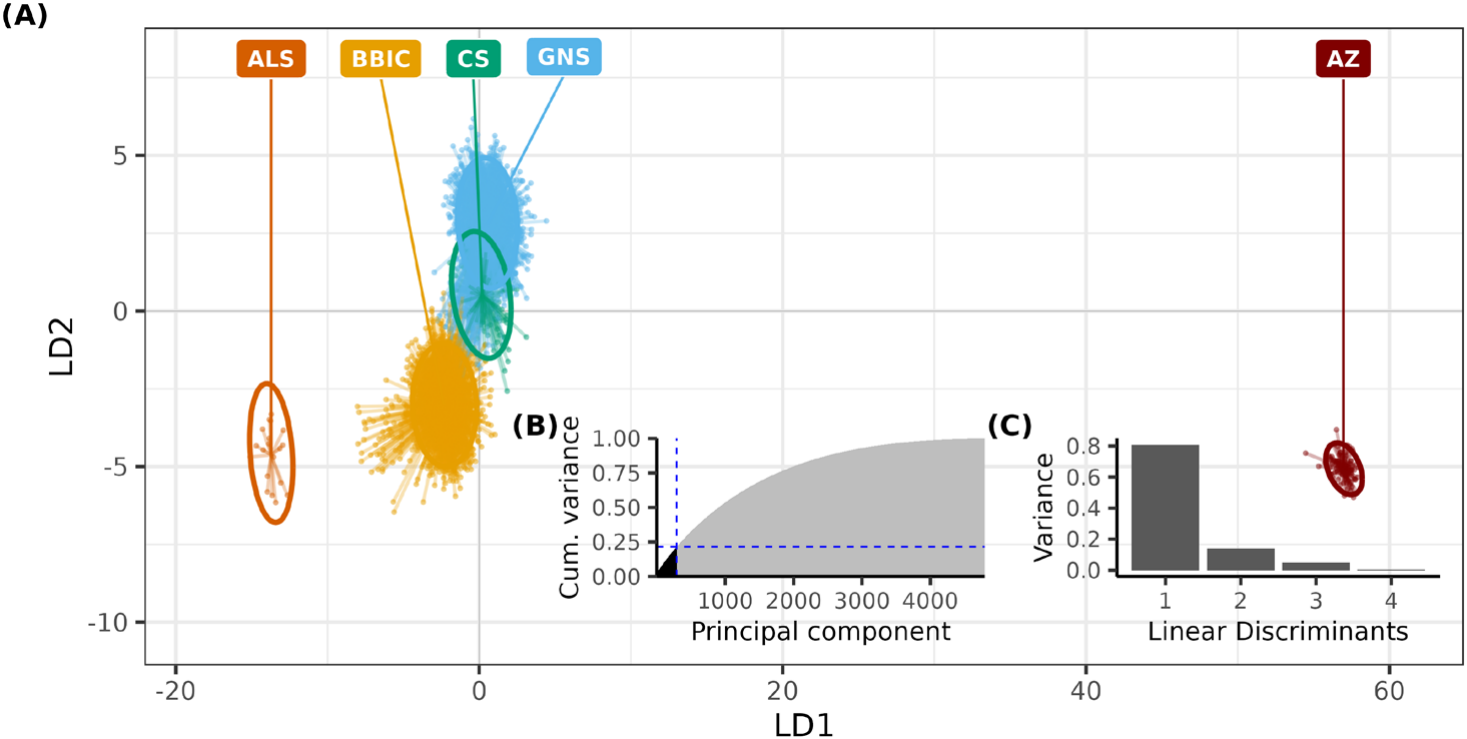
(A) DAPC with ecoregion as prior grouping for genotyped samples of *Raja clavata* , collected across the Northeast Atlantic and Mediterranean Sea. (B) Cumulative variance of optimal number of PCs retained. (C) Variance of linear discriminants (LD). For abbreviations, see Table 1.

### 3.4 DAPC With ICES Division and Coastal Zone Prior Grouping

Cross‐validation for the DAPC with ICES Division and coastal zone as prior grouping suggested retaining 230 PC axes (Figure 3B), with a mean successful assignment probability of 0.464 and a root mean squared error of 0.537. The variances explained by the DAPC's LDs are shown in Figure 3C.

**FIGURE 3:**
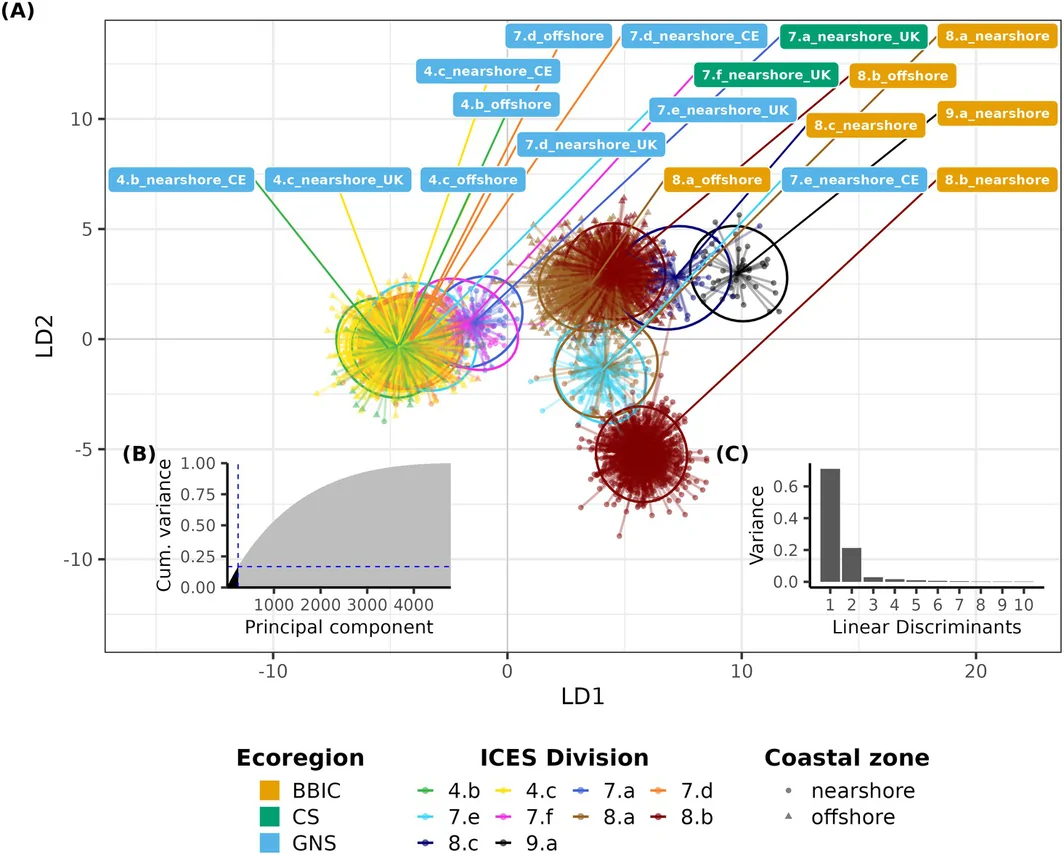
(A) DAPC for ecoregion subsets based on ICES Division and coastal zone classifications as prior grouping for genotyped samples of *Raja clavata* , collected across the Northeast Atlantic and Mediterranean Sea. (B) Cumulative variance of optimal number of PCs retained. (C) Variance of linear discriminants (LD). For abbreviations, see Table 1.

In this DAPC, samples collected in the BBIC ecoregion clustered mostly separately from samples in the GNS and CS along the first discriminant function (Figure 3A). A notable exception was represented by samples collected in *7.e_nearshore_CE*, found in the GNS but bordering the BBIC ecoregion. These samples clustered with samples collected in *8.a_nearshore* from the BBIC ecoregion (Figure 3A).

While limited separation of clusters was observed within the GNS or CS ecoregions, a notable separation of clusters was observed in the BBIC ecoregion: samples from divisions *8.a* and *8.b* clustered closely together if they came from offshore populations (*8.a_offshore* and *8.b_offshore*), while they clustered further apart from their nearshore counterparts (*8.a_nearshore* and *8.b_nearshore*) along the second LD axis, which also formed distinctly separate clusters from each other (LD2; Figure 3A).

### 3.5 DAPC De Novo Clusters

The number of clusters was evaluated by running successive *K*‐means clustering with increasing values of *K*, while retaining all 4793 PCs (Figure A4). The most plausible clustering structure was informed by the BIC values, the difference in BIC between subsequent clustering solutions and geographical and biological knowledge for this species. Both the *K* = 4 (BIC = 51,138.11) and *K* = 5 (BIC = 51,138.78) outcomes were subsequently explored.

#### 3.5.1 *K* = 4

Cross‐validation of the DAPC, with prior grouping of individuals set to four clusters (*K* = 4), suggested retaining 80 PC axes, with a mean successful assignment probability of 0.999 and a root mean squared error of 0.0015. Visualising this DAPC showed Cluster 2 (samples from AZ) clearly differentiated from the other three clusters along the first discriminant function (Figures A5A and A6A). Cluster 1 consisted primarily of samples from the GNS and CS (Figure A6A) and located close to Cluster 4 on LD1, but remained distinguishable along LD2 (Figure A5A). Cluster 3, which was composed mainly of nearshore samples collected within 30 km to the coastlines of divisions 8.a and 8.b clustered with some overlap to Cluster 4 (Figure A5A), which consisted mainly of samples collected offshore in divisions 8.a and 8.b, and from 8.c and 9.a (Figure A6A). A notable small proportion of samples collected nearshore in Division 8.a were assigned to Cluster 4, rather than Cluster 3, whereas this was not the case for samples nearshore of Division 8.b (Figures A6A and A7). All samples from the ALS were also assigned to Cluster 4 (Figures A6A and A7). Samples from the *7.e._nearshore_CE* subset were assigned mainly to Cluster 3 and, to a lesser extent, Cluster 4 (Figures A6A and A7), sharing cluster assignments with samples from the BBIC ecoregion rather than the GNS ecoregion. Samples from *7.e_nearshore_UK* in the CS ecoregion were all assigned to Cluster 1, sharing cluster assignment with the majority of samples from the GNS and CS ecoregion (Figures A6A and A7).

Depth of samples differed significantly among the inferred clusters (Table A3), with the mean depths recorded as 34 m (SD = 12) for Cluster 1, and 93 m (SD = 49) for Cluster 2 (Figure A6B). Within the BBIC ecoregion, the depths of samples from Cluster 3 (mean = 9 m, SD = 13) differed significantly (*z*‐score = 65.10, *p*‐value < 0.0001) from Cluster 4 (mean = 120 m, SD = 34; Figure A6B).

#### 3.5.2 *K* = 5

Cross‐validation of the DAPC, with prior grouping of individuals set to five clusters (*K* = 5), suggested retaining 45 PC axes, with a mean successful assignment probability of 0.999 and a root mean squared error of 0.0012. Visualising this DAPC showed Clusters 2 (samples from AZ) and 5 (samples from ALS) clearly differentiated from the other three clusters along the first discriminant function (Figure A8A; Figure 4A). Cluster 4 consisted mostly of the GNS and CS samples (Figure 4A) and clustered closely but distinctly with Cluster 3 along the second discriminant function (Figure A8A). Cluster 1, which was composed mainly of nearshore samples collected within 30 km to the coastlines of divisions 8.a and 8.b clustered with some overlap to Cluster 3 (Figure A8A), which consisted mainly of samples collected offshore in Divisions 8.a and 8.b, and from 8.c and 9.a (Figure 4A). A notable small proportion of samples collected nearshore in Division 8.a were assigned to Cluster 3, rather than Cluster 1, whereas this was not the case for samples nearshore of Division 8.b (Figure 4; Figure A9). Samples from the *7.e._nearshore_CE* subset were assigned mainly to Cluster 1 and, to a lesser extent, to Cluster 3 (Figure 4A; Figure A9), sharing cluster assignments with samples from the BBIC ecoregion rather than the GNS ecoregion. However, samples from 7.e_nearshore_UK in the CS ecoregion were all assigned to Cluster 4, sharing cluster assignment with the majority of samples from the GNS ecoregion (Figure 4A; Figure A9).

**FIGURE 4:**
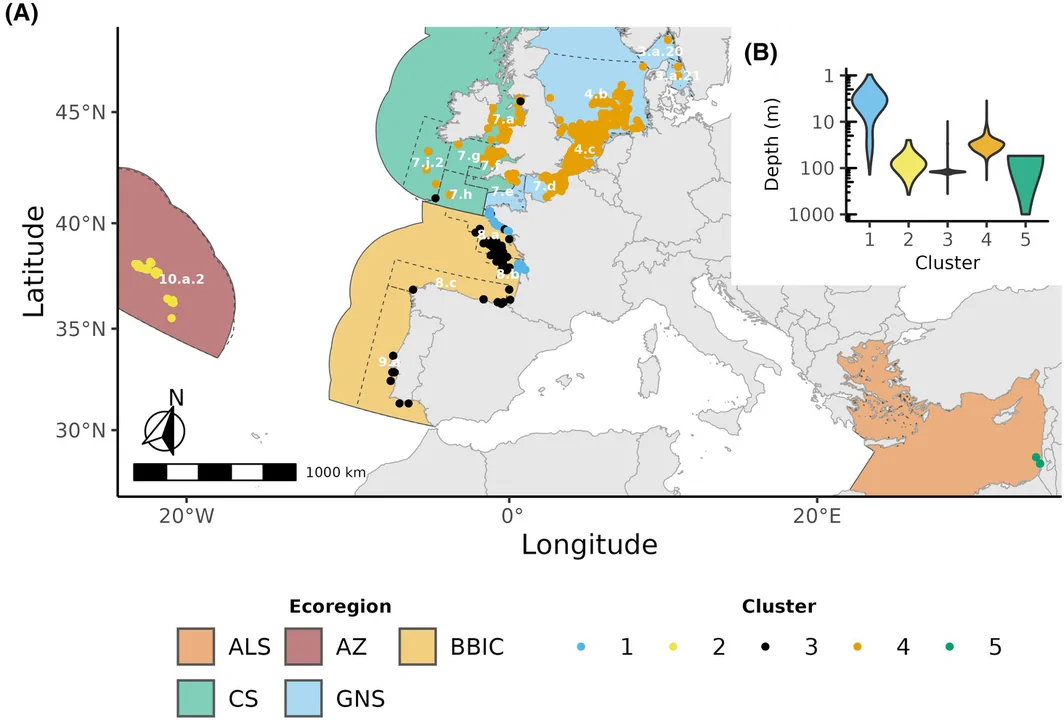
(A) Sample locations of *Raja clavata* plotted with clustering based on DAPC with de novo grouping with five clusters (*K* = 5). (B) Depth (m) of sample location for each inferred cluster, displayed as a continuous distribution. For abbreviations, see Table 1.

The depth of samples from de novo clusters set to *K* = 5 differed significantly from each other, except for Cluster 5, which is not significantly different in mean depth from Clusters 2 and 3 (Table A4). The mean depth for Cluster 5 was 153 m (SD = 296), for Cluster 2 as 93 m (SD = 49) and Cluster 4 as 34 m (SD = 12; Figure 4B). Notably, within the BBIC ecoregion, the depths of samples from Cluster 1 (mean = 9 m, SD = 13) differed significantly from Cluster 3 (mean = 120 m, SD = 21; Figure 4B) (*z*‐score = −65.11, *p*‐value < 0.001; Table A4).

Given the difference of less than one BIC unit between *K* = 4 and *K* = 5, the lack of sample coverage across the Mediterranean Sea, and the geographic distance between ALS and the other sampled ecoregions, treating ALS as a separate cluster was considered justified. This interpretation is consistent with previous research distinguishing ALS samples from those in the AZ and BBIC (Chevolot et al. 2006).

### 3.6 Admixture Analyses

#### 3.6.1 sNMF Ancestral Analysis

Cross‐entropy results for the sNMF ancestral analysis suggested five clusters (*K* = 5) as the most likely number of ancestral populations. This is indicated by the lowest cross‐entropy of 0.79586, followed by four suggested clusters (*K* = 4) with a cross‐entropy of 0.79589 (Figure A10).

No pronounced admixture was found for the AZ and ALS for the five suggested ancestral populations. The greatest ancestral cluster proportions were explained for the AZ ecoregion by Cluster 4, with an average ancestry proportion across all islands being 0.993 (Table A5). Likewise, the ALS was dominated by Cluster 3, with an ancestry proportion of 0.998 (Table A5). Admixture in the other ecoregions was more pronounced: the dominant cluster for the BBIC ecoregion was Cluster 5, with an average ancestry proportion of 0.524; for the GNS ecoregion it was Cluster 2, with an average ancestry proportion of 0.544; for the CS ecoregion Cluster 5 had an average ancestry proportion of 0.412 and Cluster 2 had an average ancestry proportion of 0.390 (Figure A11; Table A5).

Within the BBIC ecoregion, further substructure was observed. For most areas in the BBIC, the largest proportions of samples were assigned to Cluster 5. For nearshore areas in 8.a and 8.b, this was different, especially when compared to the offshore areas in the same ICES divisions. The largest proportion of samples in the 8.b_nearshore area was assigned to Cluster 1, accounting for 0.59 of the samples (Figure 5; Table A5). For the 8.a_nearshore, 0.33 of samples were assigned to Cluster 1 and 0.38 of samples to Cluster 5. In comparison, the largest proportions of offshore samples from ICES divisions 8.a and 8.b were assigned to Cluster 5, accounting for 0.60 and 0.68 of the samples, respectively (Figure 5; Table A5). Samples collected from *8.a_nearshore* showed a more similar distribution of ancestry proportions to *7.e_nearshore_CE* (0.35 for both Clusters 1 and 5), with no clear dominating clusters between Clusters 1 and 5, compared to samples collected from 8.b_nearshore, which showed clear dominance in cluster assignment to Cluster 1 (Figure A11; Table A5). No clear nearshore‐offshore differentiation was observed within other ICES divisions.

**FIGURE 5:**
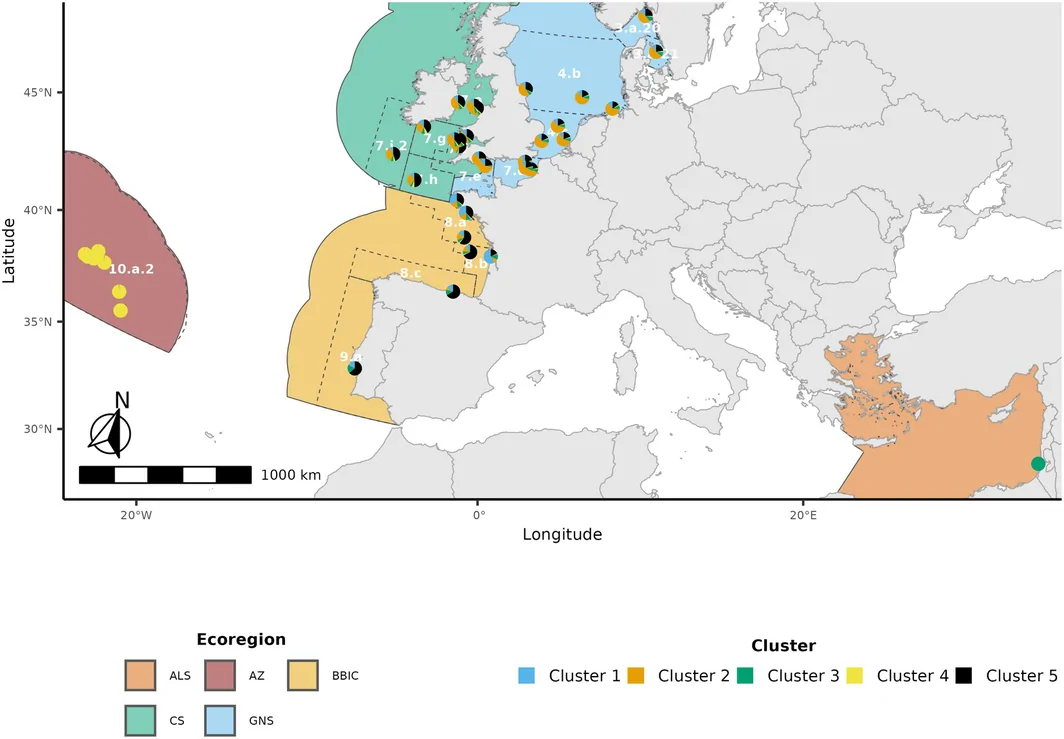
sNMF based average ancestry proportions for *Raja clavata* when classifying samples into ICES Division and coastal zone classifications for *K* = 5. Map delineates ecoregions (left legend). Colours in pie charts indicate proportion of assigned ancestry cluster for samples (right legend). For abbreviations, see Table 1.

Across the GNS, CS and BBIC ecoregions further patterns were observed: a North‐to‐South reduction of samples assigned to ancestral population Cluster 2; a Northeast‐to‐Southwest increase of samples assigned to ancestral population Cluster 5 (Figure 5). All average cluster assignment probabilities for the ICES Division and coastal zone classification can be found in Appendix A (Table A5).

Results for the model with four suggested clusters (*K* = 4) can be found in Appendix A (Figures A12 and A13; Table A6). For this model, ALS samples shared the highest proportion of ancestral population with BBIC samples for Cluster 4, while a similar pattern of admixture to the model with *K* = 5 was observed between nearshore samples of the ICES divisions 8.a and 8.b, in the BBIC, and 7.e_nearshore_CE, in the GNS, against each other and remaining samples found in the BBIC.

#### 3.6.2 ADMIXTURE Ancestral Analysis

The lowest mean cross‐validation error across 10 repetitions from the ADMIXTURE‐based ancestral analysis suggested 5 clusters (*K* = 5) as the most likely number of ancestral populations, as indicated by the lowest mean cross‐validation error of 0.563 (Figure A14; Table A7).

The ADMIXTURE results for *K* = 5 showed similar results as the sNMF ancestral analysis with *K* = 5 for proportion and distribution of ancestral clusters observed across ecoregions and ICES Division and coastal zone areas, as well as similar large and fine scale admixture patterns across the GNS, CS and BBIC ecoregions (Figures A15 and A16; Table A8).

### 3.7 Population Fixation Indices

There were 22 areas with 15 or more samples available for pairwise *F*_ST_ calculations. Pairwise *F*_ST_ values among the areas ranged from −1.07 × 10^−4^ (for *7.d_offshore* with *4.c_nearshore_CE*) to 0.55 (for Faial with ALS) (Figure 6). The largest pairwise *F*_ST_ values were observed between areas in the AZ and ALS ecoregions (> 0.5) (Figure 6). Of the 231 pairwise *F*_ST_ comparisons, 209 were statistically significant after Holm's correction. Non‐significant pairwise *F*_ST_ values were found within the CS and within the GNS ecoregion (including divisions *7.d* and *7.e*). Meanwhile, all pairwise comparisons between areas across the GNS, BBIC, AZ and ALS ecoregions, as well as among areas within BBIC, revealed statistically significant differences (Figure 6).

**FIGURE 6:**
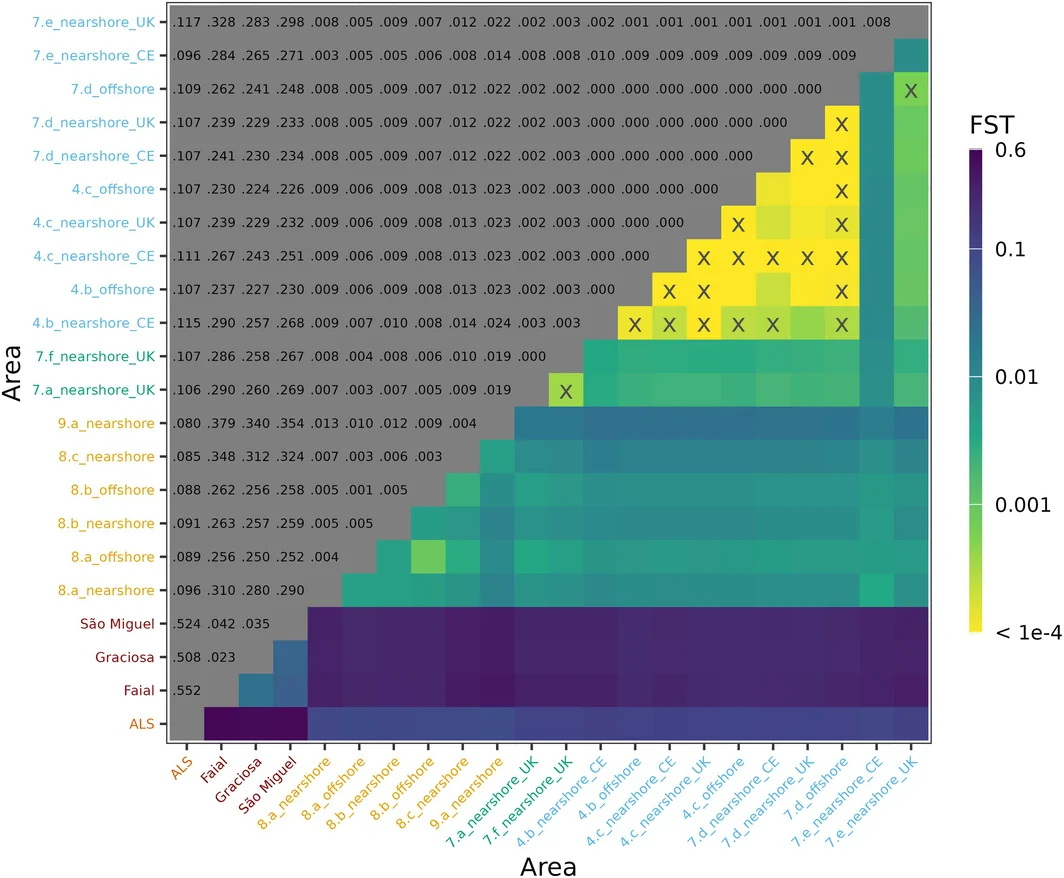
Pairwise fixation index (*F*_ST_) for *Raja clavata* across ICES Division and coastal zone classification areas from the Northeast Atlantic and Mediterranean Sea. Tiles marked with ‘X’ highlight non‐significant pairwise fixation indices (*α* > 0.05), after using Holm correction. Colours of area names represent ecoregions (orange = ALS, red = Azores, Yellow = Bay of Biscay and Iberian Coast, green = Celtic Seas, blue = Greater North Sea).

For the entire dataset, genetic differentiation was positively correlated to geographical distance (Mantel *r* = 0.56, *p* = 0.0001), consistent with isolation by distance (IBD) (Figure 7). IBD was also present within the GNS ecoregion (Mantel *r* = 0.54, *p* = 0.0030) and the BBIC (Mantel *r* = 0.79, *p* = 0.0097). *F*_ST_ values at distances < 200 km within the BBIC ecoregion were larger than those within the GNS ecoregion (Figure 7), due to the relatively larger pairwise *F*_ST_ values between nearshore and offshore samples within the Bay of Biscay (Figure 6).

**FIGURE 7:**
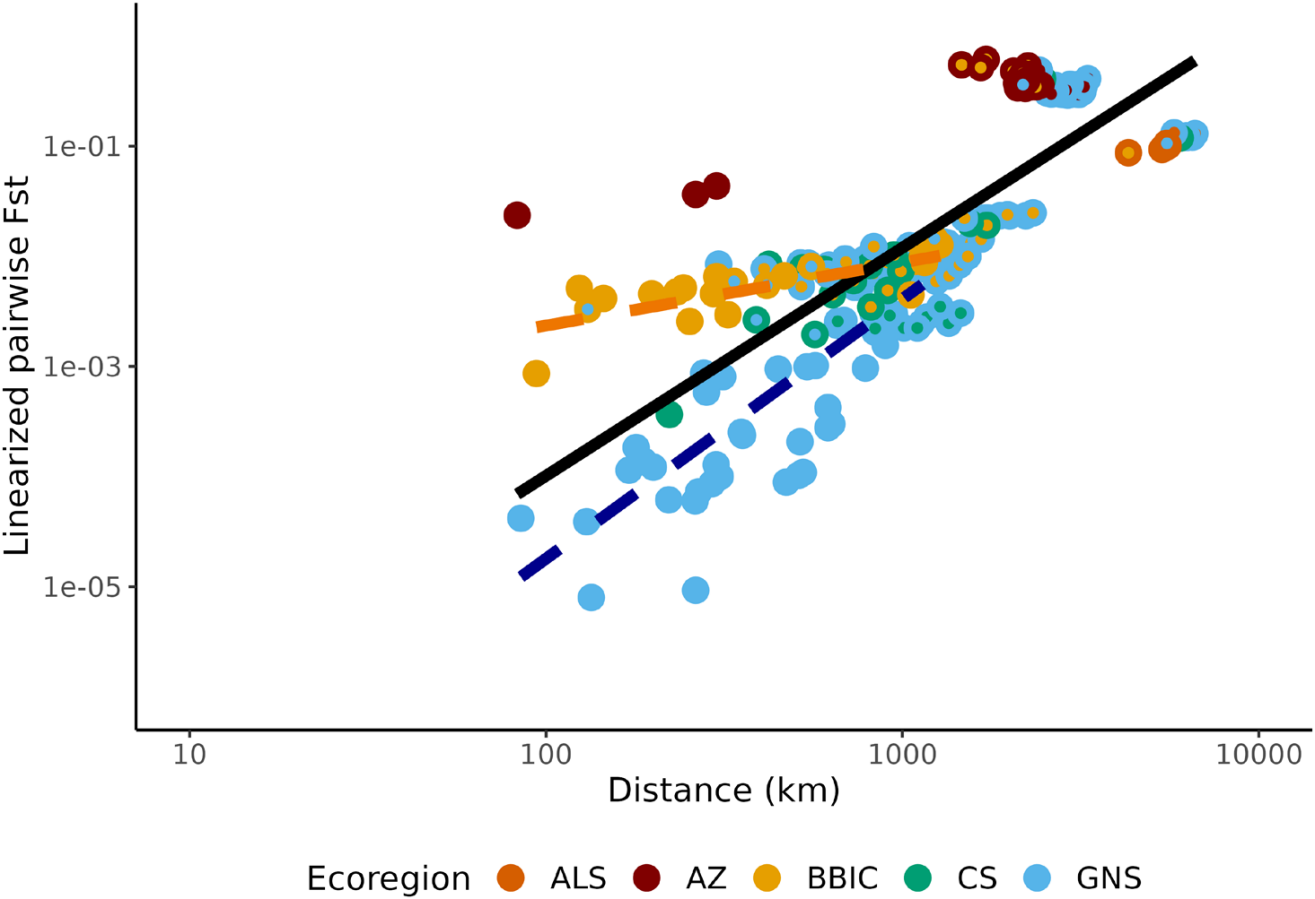
Isolation by distance analysis of linearised pairwise *F*_ST_ (Wright 1949) against log‐transformed geographical distance for *Raja clavata* , with linear regressions. Each dot represents the linearised *F*_ST_ estimate for a combination of two ICES Division and coastal zone classification areas. Ecoregions for the combinations are indicated by the fill and border colours of plotted points. Linear regression of all combinations is visualised with a black solid line. Linear regressions within the Greater North Sea and the Bay of Biscay and Iberian coast ecoregions are visualised with dashed lines in dark blue and dark orange, respectively. For abbreviations, see Table 1.

### 3.8 Genetic Diversity

Mean estimates of *H*_o_ ranged from 0.109 to 0.343 and *H*_e_ ranged from 0.107 to 0.346 (Figure 8), with no significant differences among areas within any ecoregion for either estimate (Figure A17). The lowest mean *H*_o_ and *H*_e_ were estimated for areas in the AZ ecoregion. The highest mean *H*_o_ and *H*_e_ estimates were observed for areas in the BBIC, CS and GNS ecoregions (Figure 8). Mean *F*_IS_ values were low across all areas of the ecoregions, ranging from −0.005 to 0.015 (Figure 8), with significant differences only among areas within the BBIC and GNS ecoregions (Figure A17). Overall, very low *F*_IS_ values (< 0.05) were observed across all geographic areas. The lowest *F*_IS_ values were estimated for areas in the AZ (−0.001 to 0.003) and ALS (0.001) ecoregions (Figure 8; Figure A17). Slightly higher *F*_IS_ values (mean = 0.020) were observed when combining all samples across the AZ ecoregion (Figure A18), as well as according to Cluster 2, representing AZ samples (Figure 4), of the DAPC with *K* = 5 (Figure A20). Estimated Ar across areas in the ecoregions ranged from 1.197 to 1.625, with the lowest estimates observed in areas of the AZ ecoregion and the highest estimates observed across areas in the BBIC, CS and GNS ecoregions (Figure 8), with no significant differences among areas within ecoregions (Figure A17). Significant differences for mean *H*_o_, *H*_e_ and Ar were observed across areas between ecoregions when compared against areas from AZ to ALS (Figure A17). Dunn's tests of genetic diversity estimates between ecoregions (Figure A19) and the five clusters obtained from the DAPC with *K* = 5 (Figure A21) are provided in Appendix A.

**FIGURE 8:**
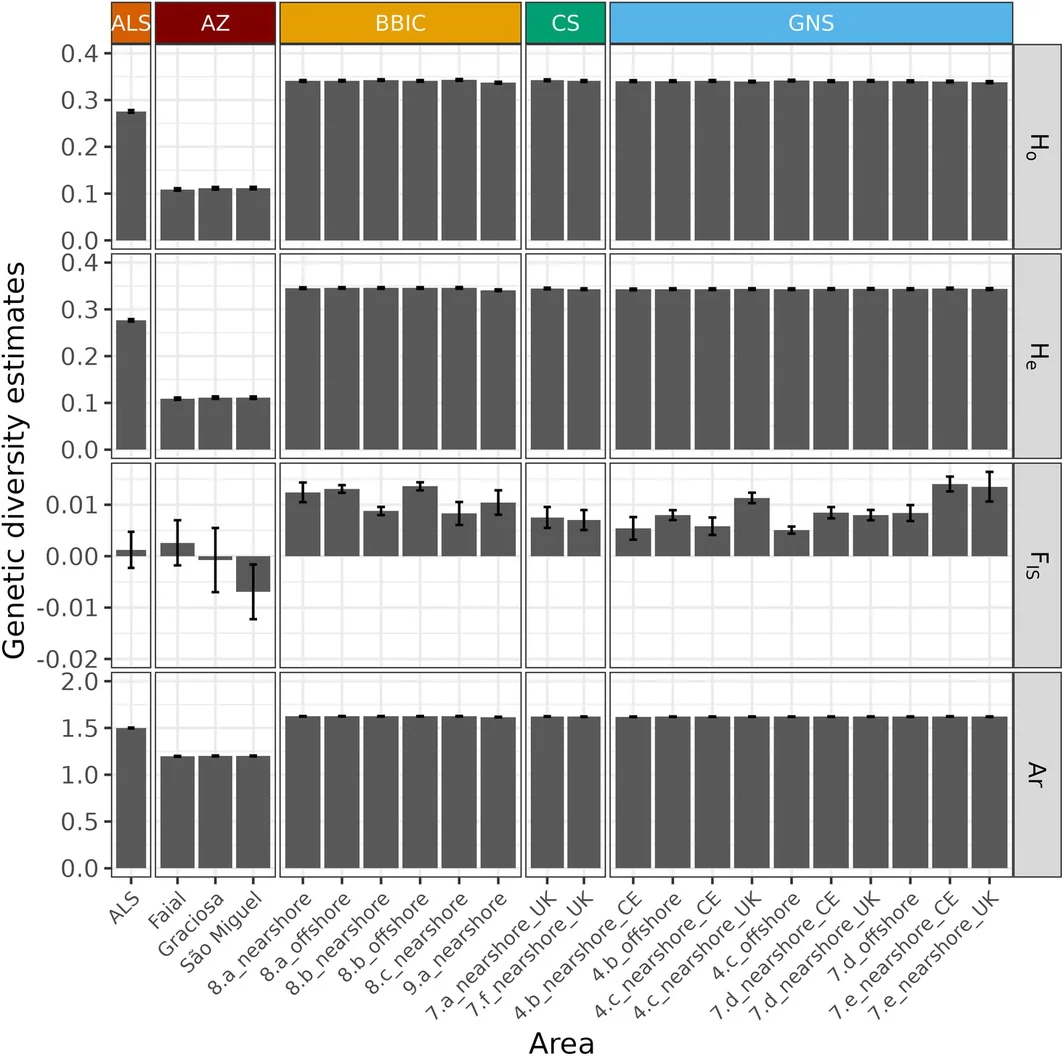
Genetic diversity estimates for *Raja clavata* . Observed Heterozygosity (*H*_o_), gene diversity (i.e., expected heterozygosity; *H*_e_), Fixation index (*F*_IS_) and allelic richness (Ar) of populations grouped by ICES Division and coastal zone classification areas, with ≥ 15 samples. Error bars indicate standard errors of means. For abbreviations, see Table 1.

## 4 Discussion

Large‐scale movements of adult skates (Rajiformes) represent their main dispersal mechanism, given their oviparous reproduction and the philopatric behaviour of reproducing females (Grubbs 2010). Adult *R. clavata* migrate annually, traversing distances of up to hundreds of kilometres, with a maximum observed straight‐line distance of 772 km (Bird et al. 2020; Hunter et al. 2005). Such migrations likely represent the main mechanism of gene flow for this species, resulting in distinct genetic population structures across varying geographic scales and barriers (Chevolot et al. 2006). This study investigated the genetic population structure of *R. clavata* using a recently develop SNP panel on a large sample set comprising more than 6900 samples spanning an in‐water geographic distance of up to 6000 km.

Our results suggest that at least five *R. clavata* subpopulations exist across the five ecoregions investigated in the Northeast Atlantic and Mediterranean Sea, with population differentiation occurring on both large (between ecoregion) and small (within ecoregion) geographic scales. The first subpopulation spanned the GNS and CS ecoregions, stretching from the East coast of Ireland to the Kattegat. Two subpopulations were identified within the BBIC: a coastal subpopulation (BBIC‐A) comprising samples from coastal waters of ICES divisions 8.a, 8.b and 7.e, and an offshore subpopulation (BBIC‐B) comprising offshore samples from ICES divisions 8.a and 8.b, and samples from ICES divisions 8.c and 9.a along the Iberian Peninsula. This finding is consistent with a close‐kin mark‐recapture study which suggested limited demographic connectivity between two distinct local populations within the Bay of Biscay (Trenkel et al. 2022). The BBIC‐B subpopulation was found much deeper than the BBIC‐A coastal subpopulation. This suggests that depth, or depth‐related gradients in environmental conditions, may act as a barrier to interbreeding, reinforcing population structure in the absence of other geographic barriers (Selkoe et al. 2008, 2010). Fishing activities are not distributed uniformly across depth gradients, and the deeper habitat occupied by the BBIC‐B subpopulation may therefore be exposed to a different exploitation regime than the BBIC‐A and GNS/CS subpopulations. Furthermore, within the coastal BBIC‐A subpopulation, further differentiation of admixture patterns and a distinct separation of clusters in the DAPC were apparent between the 8.a and 8.b nearshore populations. This small‐scale spatial population structure indicates a potential difference in their demographic history. Future studies may explore the cause of these differences and whether differences in exploitation regimes and other environmental conditions contribute to maintaining or reinforcing the observed population structure in the BBIC.

The fourth and fifth subpopulations were found in the AZ and ALS ecoregions, respectively. These results are consistent with previous studies in which mtDNA and microsatellite data showed differentiation for both AZ and ALS populations from the other ecoregions (Chevolot et al. 2006). In particular, the AZ might represent a distinct evolutionarily significant unit (Ball et al. 2016; Chevolot et al. 2006). Limited genetic differentiation was detected across the Azorean archipelago, suggesting that high levels of gene flow may occur within the Azorean archipelago, despite the distances among islands and the deep waters separating them. The history of exploitation of *R. clavata* in the Azores, like in other ecoregions, is poorly known because catches were not reported for the species separately, but for all skates and rays collectively, or more broadly as ‘elasmobranch fish’. Nevertheless, bottom trawling was always a minor part of the fishing activity and is now forbidden in the Azorean archipelago. In the Azores, thornback skate is predominantly caught by hooks and lines (ICES 2025) and not targeted by commercial fisheries. Presumably, fishing pressure in Azorean waters was lower than in other ecoregions, where persistent overcapacity of trawling fleets drove declines of demersal elasmobranch populations during the 20th century (Dulvy et al. 2000; Peristeraki et al. 2020; Sguotti et al. 2016; Stevens et al. 2000). Therefore, the effects of exploitation on the genetic diversity in the AZ might have been less pronounced than in other ecoregions, such as the Balearic Islands in the Mediterranean Sea (Ferragut‐Perello et al. 2023).

The ALS ecoregion was the only ecoregion in the Mediterranean Sea included in this study. Physical barriers such as the Strait of Gibraltar have previously been identified as a major barrier to some elasmobranchs between the Northeast Atlantic and the Mediterranean Sea (Hirschfeld et al. 2021). This barrier has also been suggested to limit dispersal of *R. clavata*, contributing to genetic differentiation between Atlantic and Mediterranean populations (Melis et al. 2023). The Mediterranean Sea comprises seven ecoregions (Spalding et al. 2007). Previous studies have suggested the presence of multiple genetic clusters of *R. clavata* in the Mediterranean Sea: one in the Levantine Sea and one in the remaining ecoregions of the central and western Mediterranean Sea (Melis et al. 2023).

Genetic diversity indicators differed substantially among areas across ecoregions. Across the BBIC and the GNS, the *F*_IS_ suggests a small deficit of heterozygotes. Within the AZ ecoregion, substantially lower estimates of heterozygosity and allelic richness were observed compared to the other ecoregions, consistent with previous phylogeographic analyses using microsatellite loci and mtDNA (Chevolot et al. 2006). The lower observed and expected heterozygosity and allelic richness in the Azores further support the interpretation that it represents a relatively small and isolated subpopulation that potentially has been affected by a strong genetic bottleneck and genetic drift (Chevolot et al. 2006; Roff 1997). The low levels of genetic diversity in the Azores relative to the other ecoregions are likely linked to limited connectivity between the AZ and the continental margin of Europe, with deep water and large geographic distances acting as barriers to dispersal and restricting gene flow (Hirschfeld et al. 2021).

Little to no evidence of inbreeding was observed across ICES Division and coastal zone areas, ecoregions or the five clusters suggested by the de novo DAPC. The ALS and three islands in the AZ showed the lowest evidence of inbreeding. However, the *F*_IS_ value calculated across all AZ samples combined was higher than the island‐specific *F*_IS_ values. Together with the low but significant *F*_ST_ values among the Azorean islands, the increased deficit of heterozygotes is consistent with a Wahlund effect. Further investigations of fine‐scale population structure within the Azores are recommended, particularly through increased sample sizes across the entire Azorean archipelago.

Significant patterns of IBD were identified using Mantel tests across all regions, and within the GNS and the BBIC ecoregions, confirming earlier findings for the species (Chevolot et al. 2006; Pasolini et al. 2011). While the sample set in this study did not allow IBD to be tested in the Mediterranean, a previous study found no significant IBD within the region (Melis et al. 2023). Within the BBIC ecoregion, mean *F*_ST_ values among geographically nearby areas were higher than expected from the overall IBD relationship, mainly because of the genetic differences between the coastal and offshore samples in the northern part of the BBIC ecoregion. To our knowledge, this study provides the first report of a genetic subdivision between a coastal and offshore population of an elasmobranch species at this spatial scale, as demonstrated by the apparent genetic differentiation between the BBIC‐A and BBIC‐B subpopulations of *R. clavata* . Previous studies have found distinct coastal and offshore genetic populations in the Northeast Atlantic, such as for exploited teleosts, with some genetic variations being potentially adaptive (Berg et al. 2021; Johansen et al. 2020; Knutsen et al. 2018; Michalsen et al. 2014).

Genetic connectivity studies are important for defining appropriate units for stock assessments and for informing fisheries management and conservation planning (Jorgensen et al. 2022; Reiss et al. 2009; Waples et al. 2008). ICES currently considers eight assessment units for *R. clavata* in the Northeast Atlantic. These units are based on ecoregion boundaries, discontinuities in geographical distribution of species, tagging information and expert knowledge (Marandel et al. 2018). Meanwhile, commercial catches of skates and rays in EU and UK waters are managed under five zones that are not aligned consistently with the ICES units. Both the ICES assessment framework and the EU/UK catch‐management framework contain more spatial units than the four genetically distinct groups observed in this study outside the Mediterranean. Assessment and management units are usually defined as demographically independent populations governed by local birth and death rates rather than by migration (Palsbøll et al. 2007). Linking demographic independence to an appropriate threshold level of population genetic divergence requires establishing species‐specific relationships between demographic characteristics and population genetic dynamics. These relationships are influenced by relative population abundance and by dispersal rates and patterns (Marandel et al. 2018). Modern techniques, such as close‐kin mark‐recapture (Trenkel et al. 2022) for population abundances and tagging studies (Hunter et al. 2005) for dispersal rates, may help stock delineation (Cadrin et al. 2023). As an example of a discrepancy between assessment units or ecoregions and distinct genetic subpopulations, our analysis showed that samples from the 7.e nearshore coastal zone (*7.e_nearshore_CE*), located in the GNS and bordering the CS and BBIC, are genetically similar to BBIC nearshore samples. Clearly the revision of ICES assessment units for *R. clavata* will not be initiated without quantification of genetic and demographic metapopulation structure. Moreover, policy makers rightfully would be reluctant at revising management units without scientifically sound basis, which this study contributes to providing. Nevertheless, to be practical, assessment and management units should be sufficiently large (Hilborn and Walters 1992). Availability of data constrains the spatial scale at which assessments are achievable. For example, abundance indices required for population dynamics modelling may not be available for every local population whilst management may need to align with jurisdictional areas and consider the practicality of enforcement. The question is whether individual genetic components of a stock may decline below levels at which genetic diversity is lost under current management practices. Examples of historical overexploitation resulting in loss of genetic diversity can be found in the Balearic Islands for *R. clavata* (Ferragut‐Perello et al. 2023) and for other skate species (Ferragut‐Perello et al. 2026).

The management and conservation of this species will benefit from continued improvements in our understanding of its global genetic population structure. This study contributes by expanding beyond mitochondrial DNA and microsatellites to a high‐density nuclear SNP dataset across Northeast Atlantic ecoregions. The spatial resolution of the dataset enabled the detection of fine‐scale boundaries between distinct genetic subpopulations within the Northeast Atlantic. These results provide valuable information for future conservation management strategies. Additional samples from regions not covered in this study should be collected and used to supplement the current dataset. Priority regions include the northern parts of the Greater North Sea and Celtic Sea, the Balearic Islands, Aegean Sea, Ligurian‐Tyrrhenian area (Ferragut‐Perello et al. 2023), Black Sea, the Atlantic and Mediterranean coasts of Africa (Stehmann and Bürkel 1984) and the Southwestern Indian Ocean (Stehmann 1995). Genotyping existing and newly collected specimens from these regions using this SNP chip may also aid in improved understanding of the spatial distribution of *R. clavata* , resolving misidentification and taxonomic uncertainty involving other skate species such as *Raja straeleni* (Poll 1951) and *Raja maderensis* (Serra‐Pereira et al. 2010), which are still common (Crobe et al. 2021; Ebert and Stehmann 2013).

Future studies using the same SNP panel allow direct integration of new genotypes into the dataset used in this study. Alternatively, resequencing data could be compared with the current SNP dataset. The archived tissue samples collected decades ago and analysed in this study demonstrate the value of having sample repositories through biobanking. Such biobanks provide opportunities to infer genetic changes over time and assess the effects of environmental pressures (Chen and Mastromonaco 2025). Although biobanking is becoming increasingly widespread, geographical and taxonomic gaps remain. A lack of sample availability for sharks, rays and skates highlights bias in current efforts (Chen and Mastromonaco 2025). As whole‐genome sequencing becomes increasingly affordable, and as reference genomes for *R. clavata* continue to develop, archived samples may provide increasingly unbiased estimates of genome‐wide genetic diversity.

The SNP panel applied in this study was not selected to include variation known to be under selection. Future NGS resequencing of *R. clavata* populations will capture functional variation, including loci potentially under selection. Rich genome‐wide datasets can reveal recent demographic developments, for example, resulting from fisheries pressure, and reveal how populations adapt to changing ecological conditions, such as those expected under climate change and habitat fragmentation. Other demersal species in various marine regions are suspected to have changed life‐history characteristics under the strong length‐dependent mortality induced by fishing. These changes in life history include earlier maturation and reduced growth rates of adults (Jørgensen et al. 2007). Age determination, and thus age‐at‐maturation and growth, are notoriously difficult for elasmobranchs that lack scales and otoliths (Cailliet et al. 1986; Greenway et al. 2026). Genotyping loci associated with growth and maturation in specimens collected before industrial fishing started may shed light on how fishing induces evolution. Where historic material suitable for sequencing is unavailable, space‐for‐time substitution, contrasting areas with different fishing pressures, may provide insight into evolutionary responses.

To conclude, this study represents an extensive and detailed sampling effort across the Northeast Atlantic and is among the largest population genomic studies conducted for an elasmobranch species to date. It provides high‐resolution insights into the commercially important thornback skate, *Raja clavata* , and demonstrates strong genetic evidence for: (1) population structure across both large and small spatial scales in the Northeast Atlantic and Mediterranean Sea; (2) strong patterns of IBD across the Northeast Atlantic, and within the GNS and the BBIC ecoregions; (3) distinct genetic differentiation between offshore and nearshore populations in the Bay of Biscay, and evidence for fine‐scale genetic differences among nearshore subpopulations; and (4) lower genetic diversity in the Azores, without strong evidence of elevated inbreeding in any ecoregion. The rich SNP dataset obtained and shared publicly will provide a valuable resource for future studies to extend population genetic research on *R. clavata* geographically, longitudinally and functionally.

## Author Contributions

**Timo Michael Staeudle:** conceptualization (lead), data curation (lead), formal analysis (lead), investigation (equal), methodology (lead), project administration (lead), resources (equal), software (lead), visualization (lead), writing – original draft (lead), writing – review and editing (lead). **Eleanor S. I. Greenway:** data curation (equal), resources (equal), writing – review and editing (equal). **Jurgen Batsleer:** funding acquisition (equal), resources (equal). **Cristina Rodríguez‐Cabello:** resources (equal), writing – review and editing (equal). **Diana Catarino:** resources (equal), writing – review and editing (equal). **Jim R. Ellis:** resources (equal), writing – review and editing (equal). **Eva Giacomello:** resources (equal), writing – review and editing (equal). **Pascal Lorance:** data curation (equal), writing – review and editing (equal). **Hendrik‐Jan Megens:** supervision (supporting), writing – review and editing (equal). **Joey J. Portier:** data curation (supporting), writing – review and editing (equal). **Nir Stern:** resources (equal), writing – review and editing (equal). **Klaas Sys:** resources (equal), writing – review and editing (equal). **Jan Jaap Poos:** funding acquisition (lead), supervision (lead), writing – original draft (equal), writing – review and editing (equal).

## Funding

This project received funding from the Dutch Ministry of Agriculture, Nature and Food Quality, under the umbrella of the ‘Bridging knowledge gaps for sharks and rays in the North Sea’ project and the European Maritime and Fisheries Fund, under the umbrella of the ‘OSW2.1’ project for scientific collaboration between the fishing industry and research. Samples from the Kattegat‐Skagerrak were taken as part of project ‘Utveckling av förvaltning av torsk i Kattegatt (Developing management of Kattegat cod)’, funded by the Swedish Agency for Marine and Water management (SwAM) contract no. Dnr 1049‐20, project. Samples from the Bay of Biscay were collected as part of project GenoPopTaille, contract ANR‐14‐CE02‐0006‐01, funded by the French National Research Agency. Azorean samples were collected under the ARQDAÇO monitoring surveys funded by the Regional Government of the Azores. DC was funded through the FCT—Foundation for Science and Technology, I.P., under the project UIDP/05634/2020 granted to OKEANOS, and through the Marine Beacon project, funded by the European Union's Horizon Europe research and innovation programme under grant agreement no. 101135237. E.G. was funded by CONDOR/DEMERSAIS projects (Regional Government of the Azores). OKEANOS received national funds through the FCT, under the project UIDB/05634/2023 and UIDP/05634/2023 and through the Regional Government of the Azores through the project M1.1.A/FUNC.UI&D/003/2021‐2024. P.L. was funded through the European Union's Horizon 2020 research and innovation programme under the grant agreement number 773713 (PANDORA).

## Disclosure

*Benefits Generated*: A research collaboration was developed with scientists from the countries providing samples or data for genotyping, co‐authorship was offered, additional benefits from this research accrue from the sharing of our data, code and results on public databases as described above. The research addresses a priority concern: the conservation of organisms being studied.

## Ethics Statement

The majority of fin clips were collected from dead animals landed by commercial fishers and intended for human consumption. Dutch, Belgium and Spanish samples collected from live animals on board of commercial and research vessels were collected as part of the InnoRays project under the animal experiment number 2018.D‐0006.001 and in accordance with institutional and national regulations. Azorean samples were collected under the Azorean permit CCIR‐RAA/2023/16. Swedish samples were collected under the Swedish ethical permit ID 002979; Dnr 5.8.18‐06684/2020 and followed the national legal requirements. Samples provided by the United Kingdom were collected offshore or from dead animals in accordance with CEFAS institutional protocols. The majority of Israelian samples were collected from confiscation event of illegal catches, while a small set of three samples were collected from deep sea monitoring cruises following national legal requirements, and subsequently send to and provided to us by the Steinhardt Museum of Natural History for further analyses. Genotyping data for French samples were provided from previous projects, for which sample collection and genotyping was obtained in compliance with legal requirements in France.

## Conflicts of Interest

The authors declare no conflicts of interest.

## Figures and Tables

**FIGURE A1:**
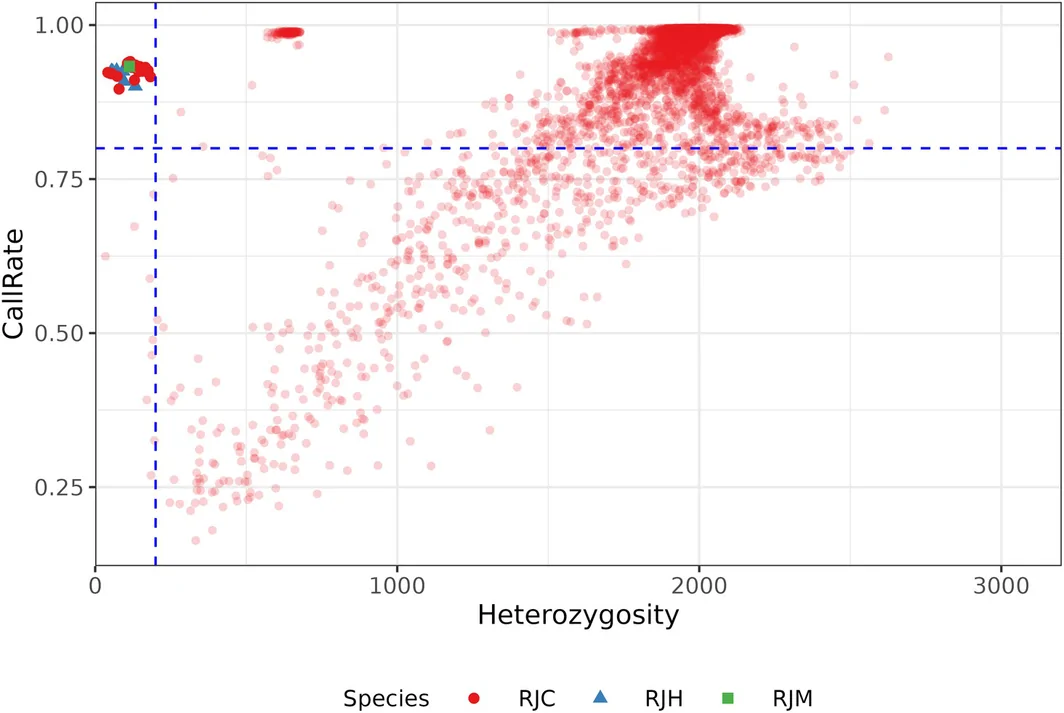
Sample call rate and locus heterozygosity of samples collected from the Northeast Atlantic and the Mediterranean Sea. Blue dashed lines indicate call rate (≥ 0.8) and heterozygosity (< 200 SNPs) thresholds used to identify outlier (likely misidentified) *Raja clavata* (RJC) samples, clustering with control samples of *Raja brachyura* (RJH) and *Raja montagui* (RJM).

**FIGURE A2:**
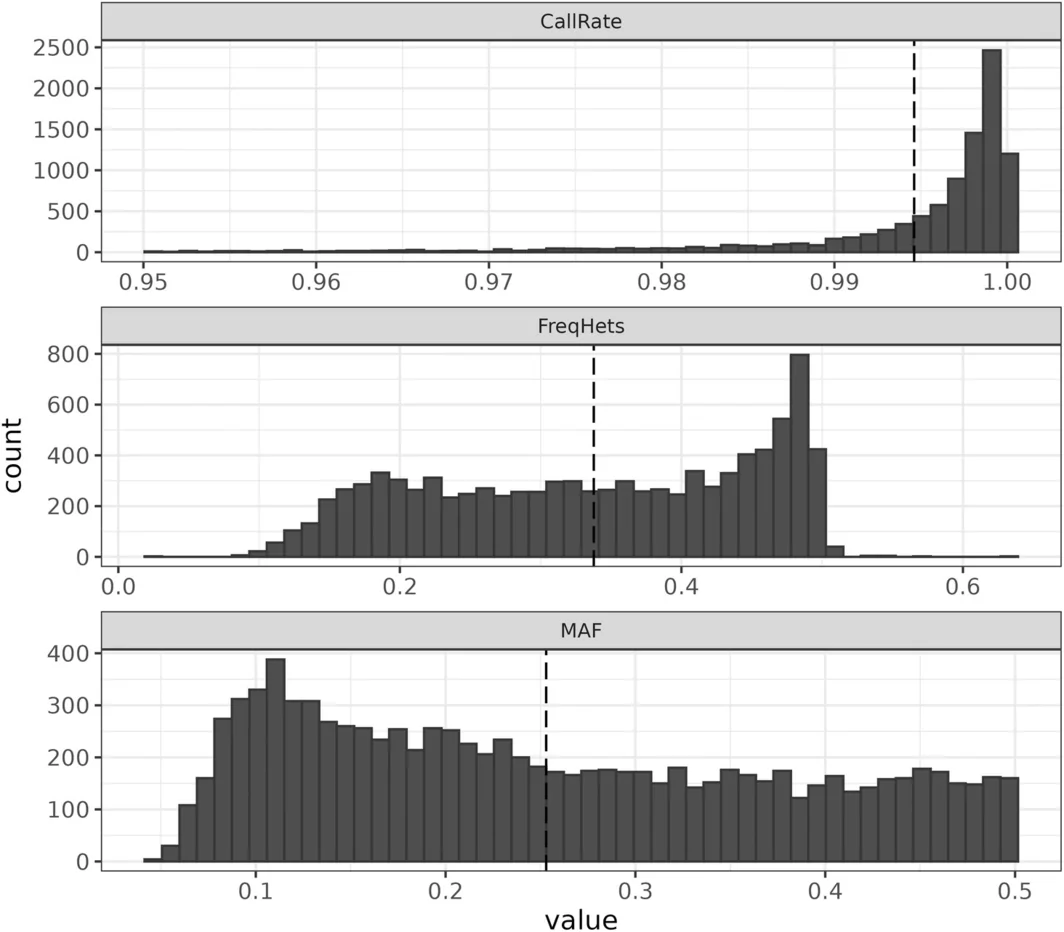
Loci call rate (CallRate), loci heterozygosity (FreqHets) and Minor Allele Frequency (MAF) after final filtering step, with 6902 genotyped samples of *Raja clavata* collected from the Northeast Atlantic and Mediterranean Sea, and 4793 loci. Dashed black lines indicate the mean of each statistic.

**FIGURE A3:**
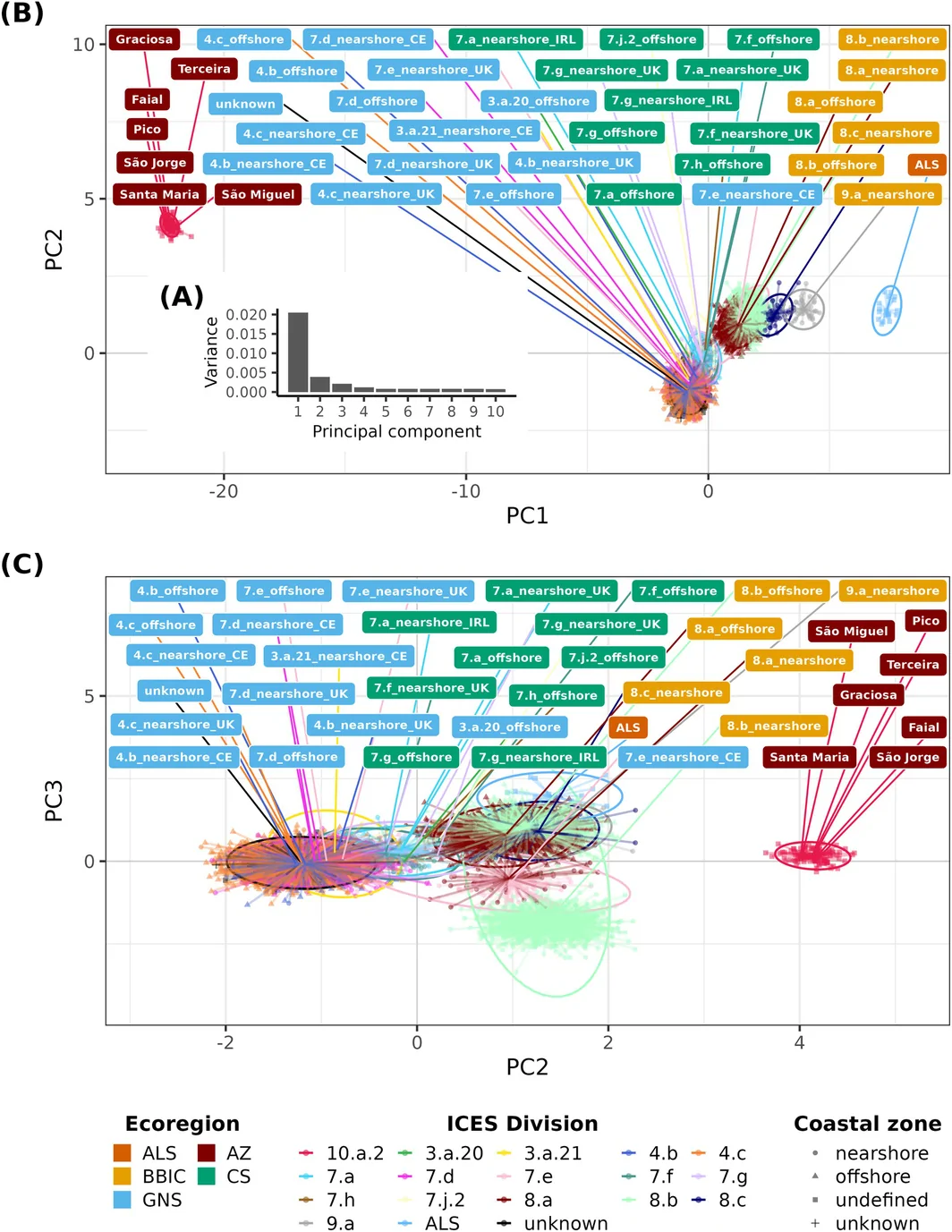
Principal component analyses (PCAs) for genotyped samples of *Raja clavata* , collected from the Northeast Atlantic and Mediterranean Sea. (A) Variance of principal components (PC). (B) PC1 and PC2. (C) PC2 and PC3. For abbreviations, see Table A1.

**FIGURE A4:**
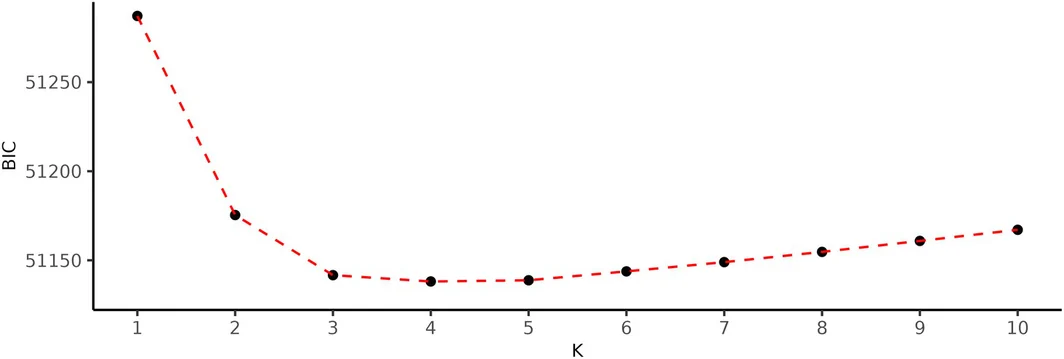
Evaluation of DAPC without prior using Bayesian Information Criterion (BIC) for genotyped samples of *Raja clavata* , collected from the Northeast Atlantic and Mediterranean Sea.

**FIGURE A5:**
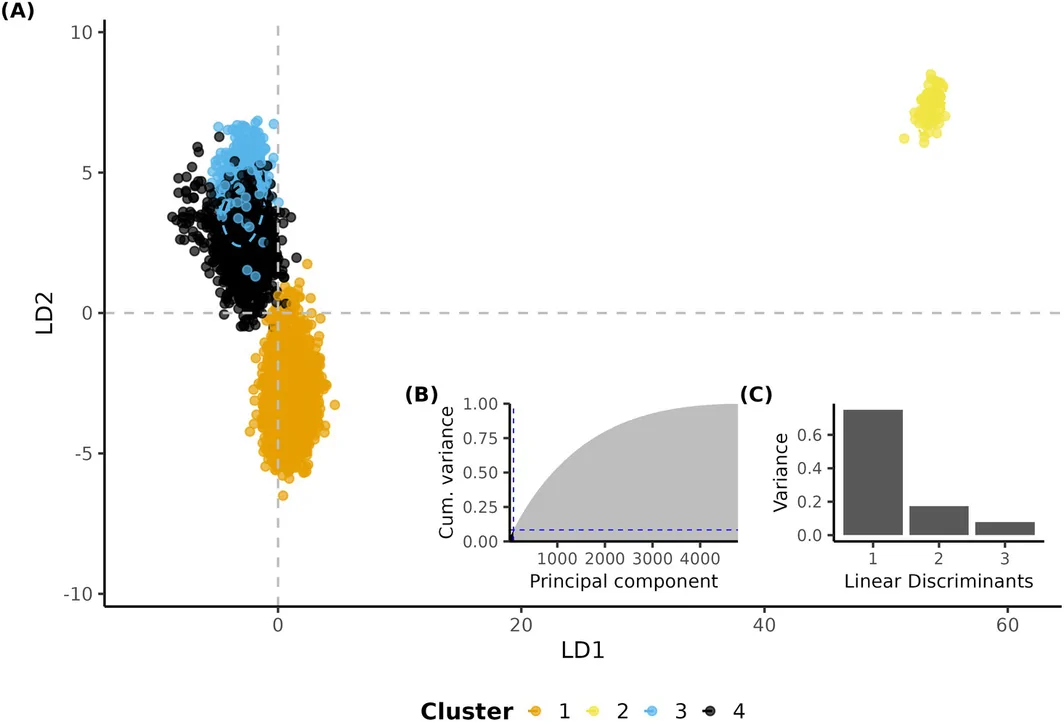
(A) DAPC without prior and grouping based on *K* = 4, for genotyped samples of *Raja clavata* , collected across the Northeast Atlantic and Mediterranean Sea. (B) Cumulative variance of optimal number of PCs retained. (C) Variance of linear discriminants (LD).

**FIGURE A6:**
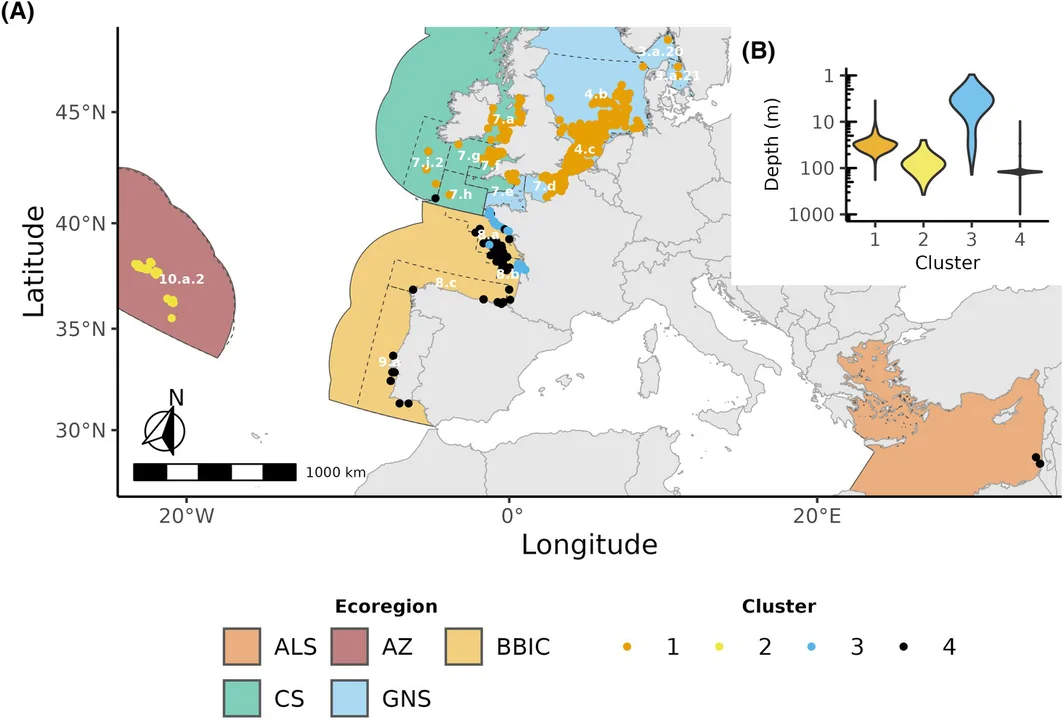
(A) Samples of *Raja clavata* plotted by four inferred clusters (*K* = 4) from discriminant analysis of components (DAPC). (B) Depth (m) of sample location for each inferred cluster, displayed as a continuous distribution. For abbreviations, see Table A1.

**FIGURE A7:**
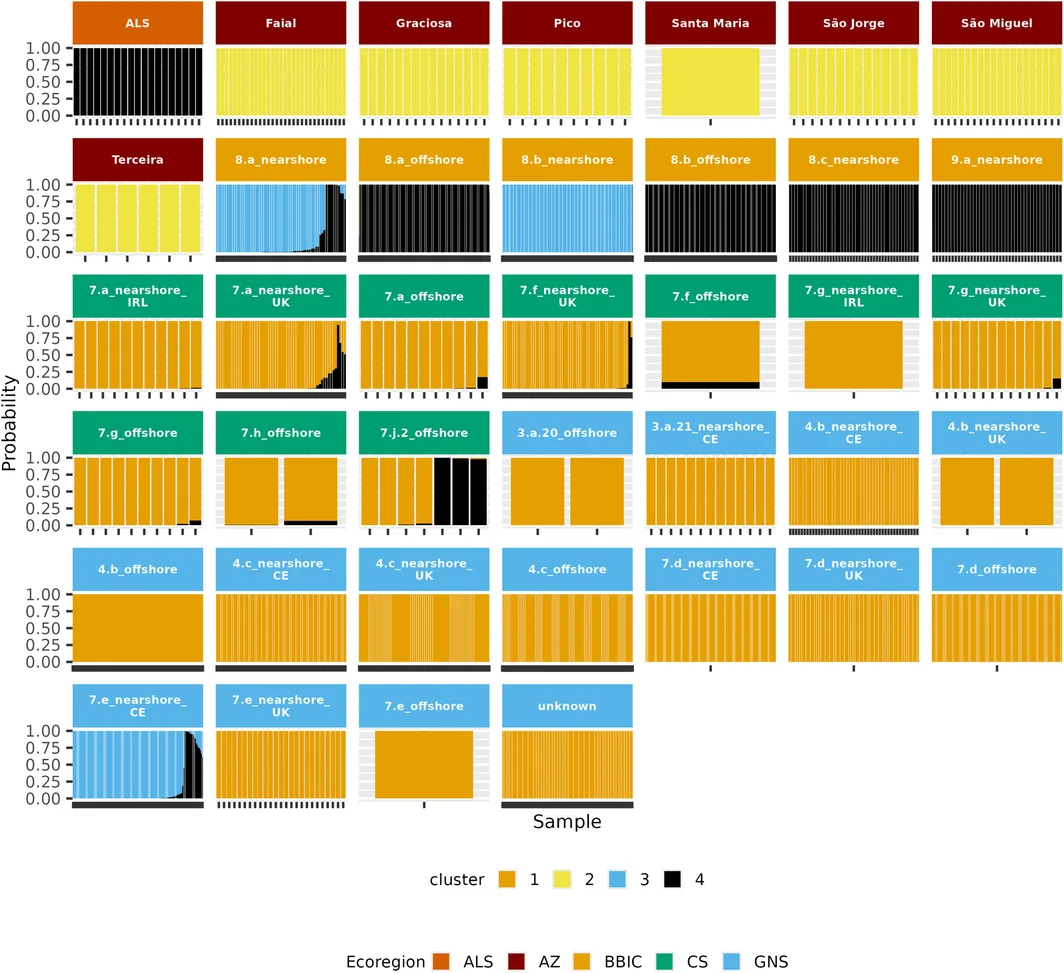
Assigned cluster probabilities for individuals from DAPC without prior and grouping based on four clusters (*K* = 4), for genotyped samples of *Raja clavata* . Bar colours indicate assigned cluster probabilities for each sample. Facet colours indicate ecoregion. For abbreviations, see Table A1.

**FIGURE A8:**
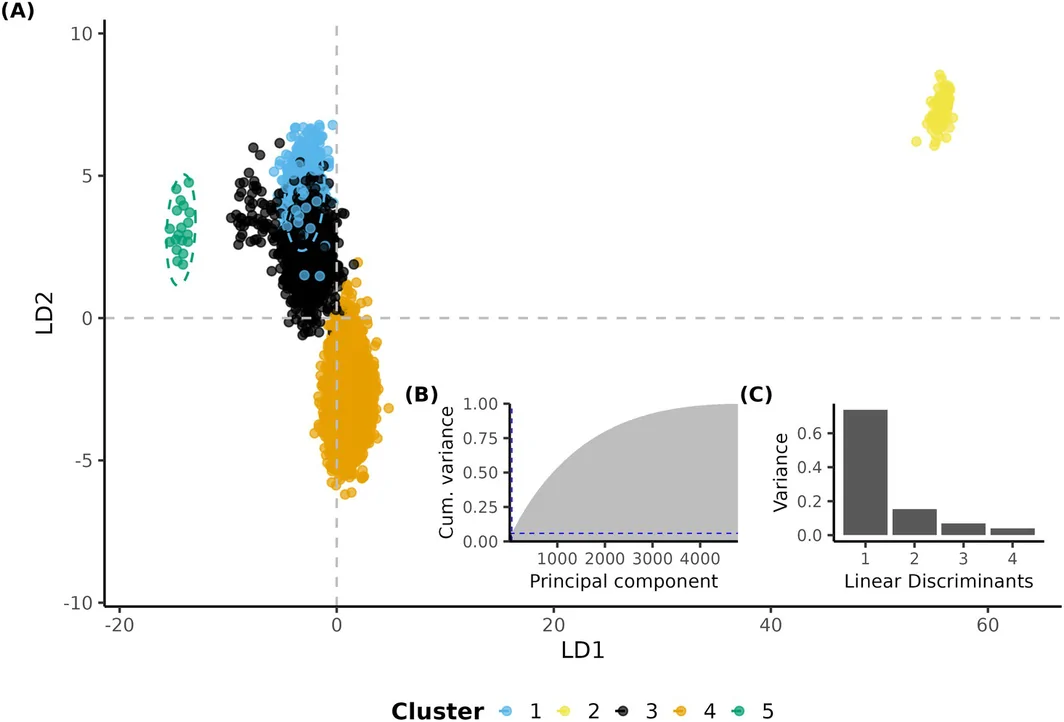
(A) DAPC based on de novo grouping with five clusters (*K* = 5) for genotyped samples of *Raja clavata* , collected across the Northeast Atlantic and Mediterranean Sea. (B) Cumulative variance of optimal number of PCs retained. (C) Variance of linear discriminants (LD).

**FIGURE A9:**
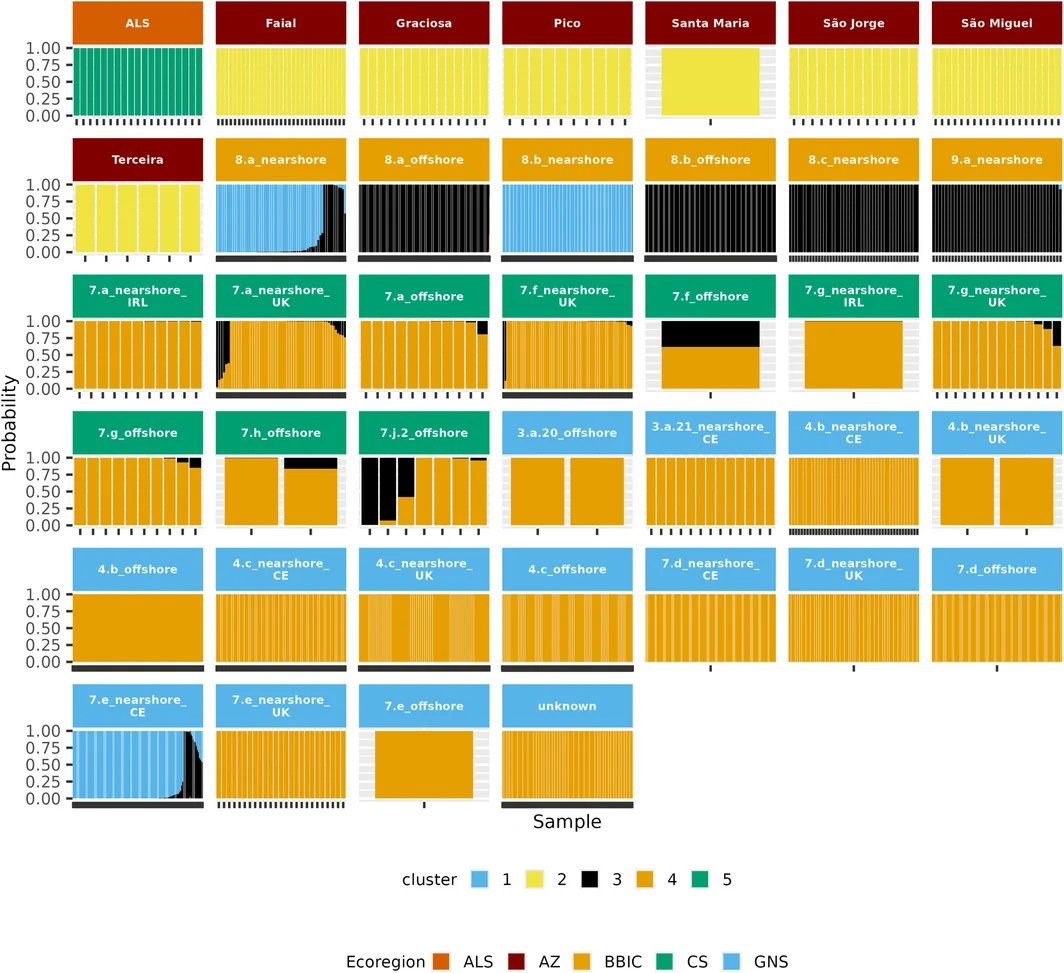
Assigned cluster probabilities for individuals from DAPC without prior and grouping based on five clusters (*K* = 5), for genotyped samples of *Raja clavata* . Bar colours indicate assigned cluster probabilities for each sample. Facet colours indicate ecoregion. For abbreviations, see Table A1.

**FIGURE A10:**
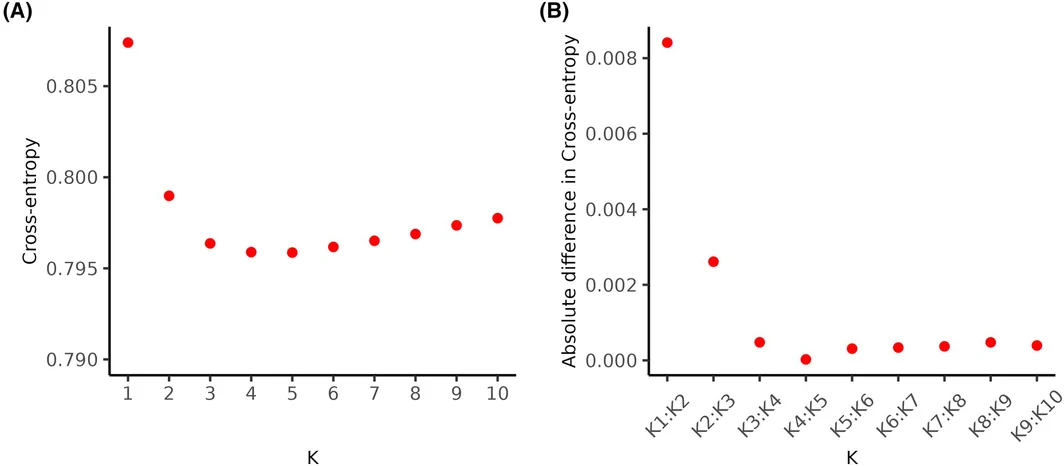
Cross‐entropy (A) of clusters (*K*) and absolute difference of cross‐entropy between subsequent clusters (B) from sNMF based admixture analyses, for genotyped samples of *Raja clavata* , collected across the Northeast Atlantic and Mediterranean Sea.

**FIGURE A11:**
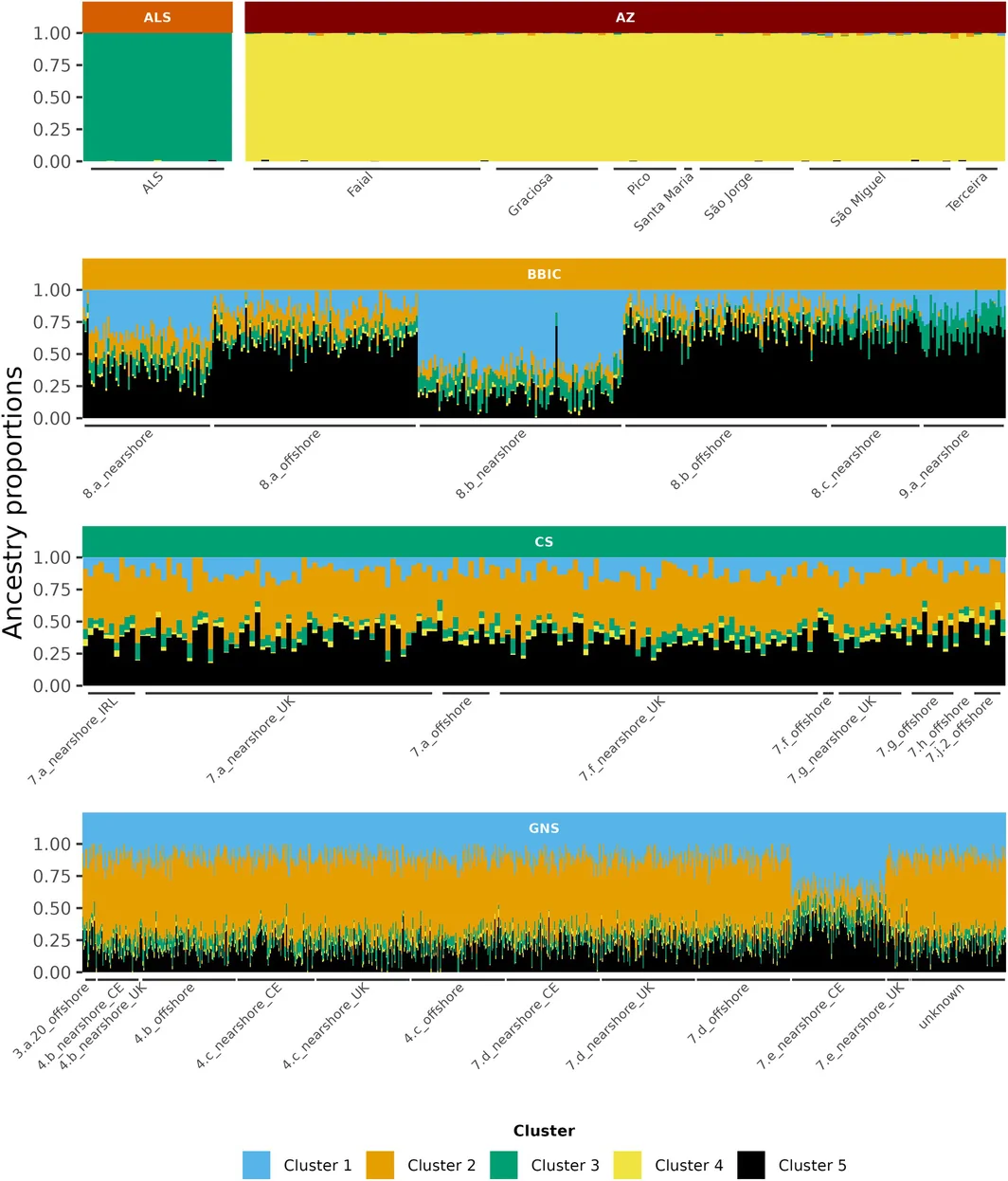
sNMF based ancestry proportions per *Raja clavata* sample, assuming five ancestral clusters (*K* = 5). Results are grouped by ecoregion, and within ecoregion subsets based on ICES Division and coastal zone classification, indicated by horizontal bars below stacked bar charts. For clarity, a random sub‐sample of up to 100 individuals per within ecoregion subset is displayed. For abbreviations, see Table A1.

**FIGURE A12:**
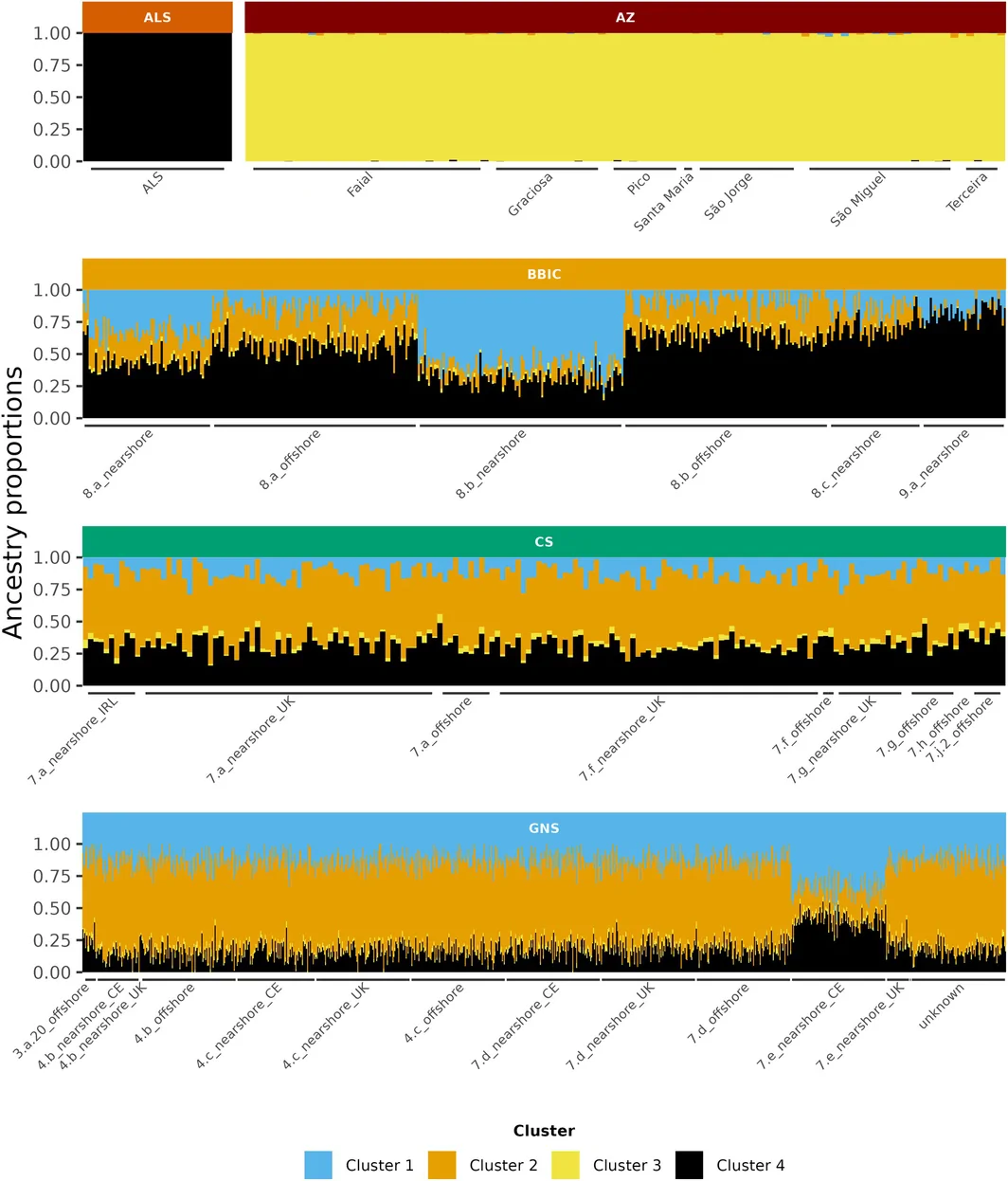
sNMF based ancestry proportions per *Raja clavata* sample, assuming four ancestral clusters (*K* = 4). Results are grouped by ecoregion, and within ecoregion subsets based on ICES Division and coastal zone classification, indicated by horizontal bars below stacked bar charts. For clarity, a random sub‐sample of up to 100 individuals per within ecoregion subset is displayed. For abbreviations, see Table A1.

**FIGURE A13:**
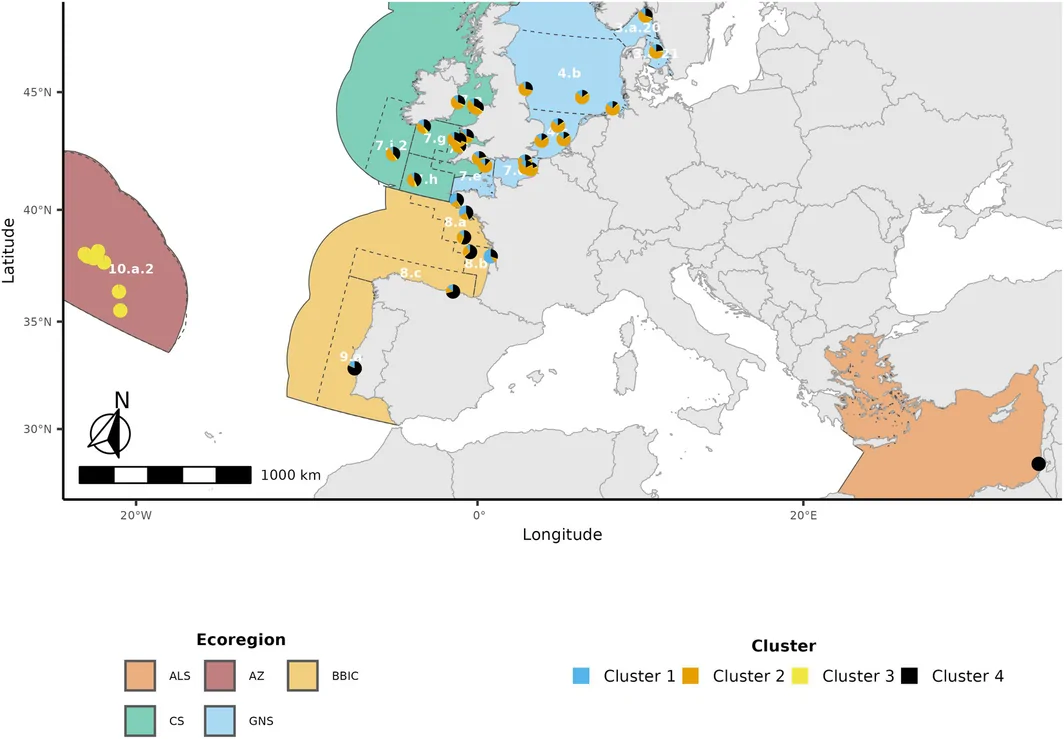
sNMF based average ancestry proportions of *Raja clavata* samples when classifying samples into ICES Division and coastal zone classifications for *K* = 4. Map delineates ecoregions (left legend). Colours in pie charts indicate proportion of assigned ancestry cluster for samples (right legend). For abbreviations, see Table A1.

**FIGURE A14:**
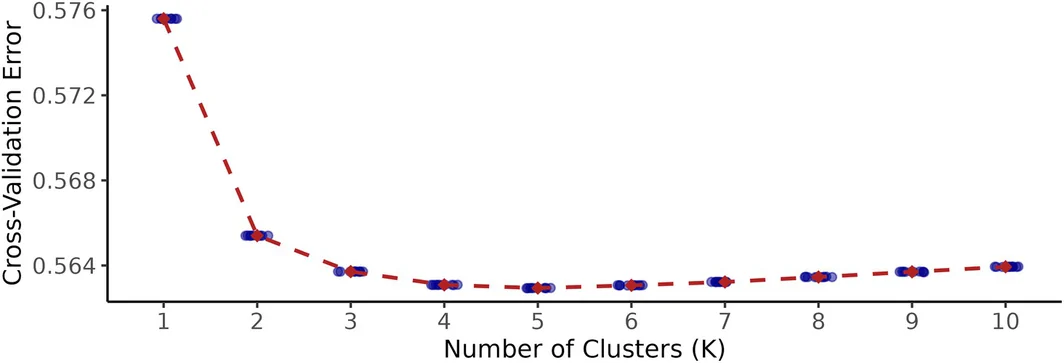
Cross‐validation error of clusters (*K*) from ADMIXTURE based admixture analyses, for genotyped samples of *Raja clavata* , collected across the Northeast Atlantic and Mediterranean Sea. Blue dots indicate Cross‐validation error of ten replicates per *K*. Red line and symbol indicates mean cross‐validation error of replicates per *K*.

**FIGURE A15:**
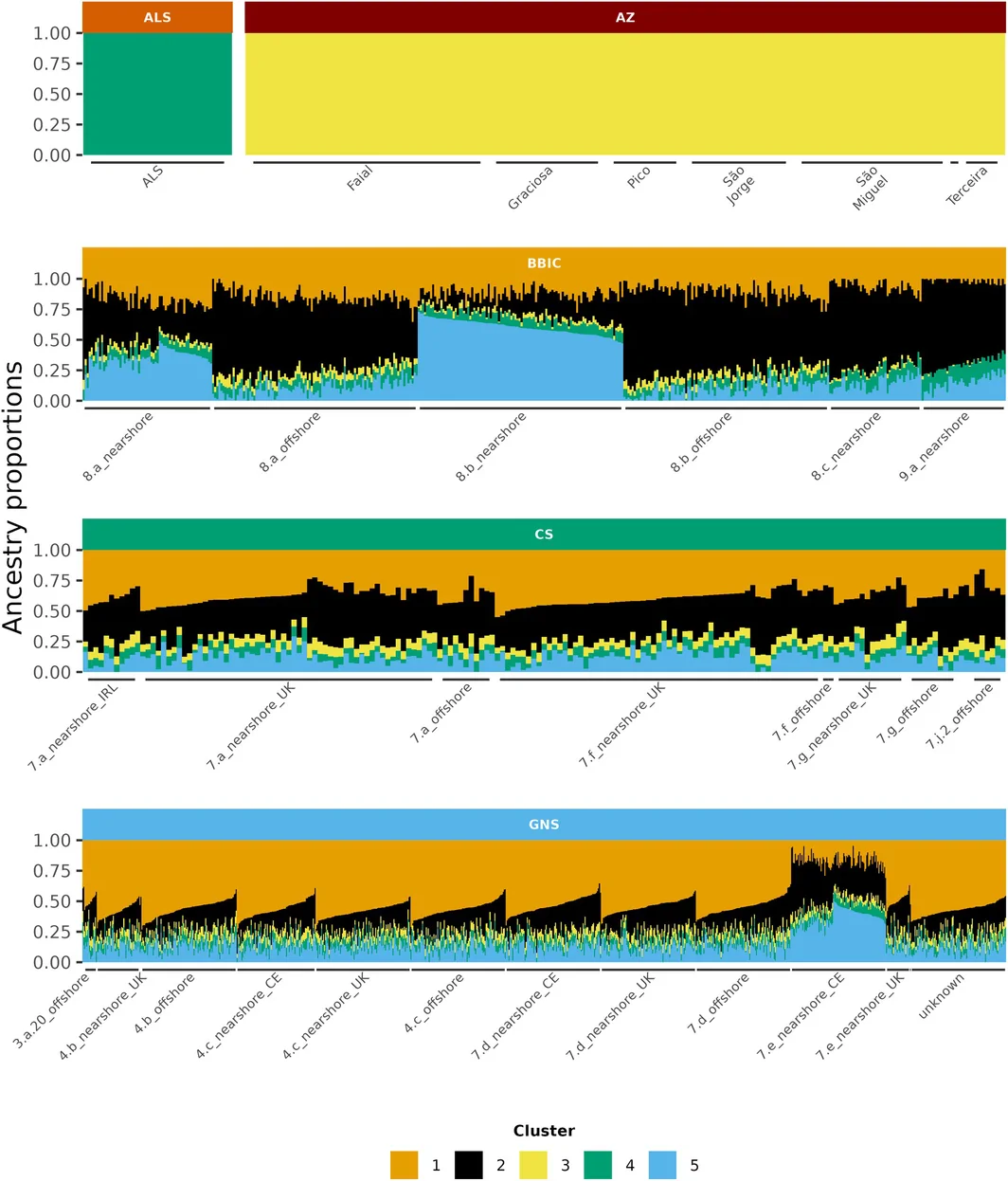
ADMIXTURE based ancestry proportions per *Raja clavata* sample, assuming five ancestral clusters (*K* = 5). Results are grouped by ecoregion, and within ecoregion subsets based on ICES Division and coastal zone classification, indicated by horizontal bars below stacked bar charts. For clarity, a random sub‐sample of up to 100 individuals per within ecoregion subset is displayed. For abbreviations, see Table A1.

**FIGURE A16:**
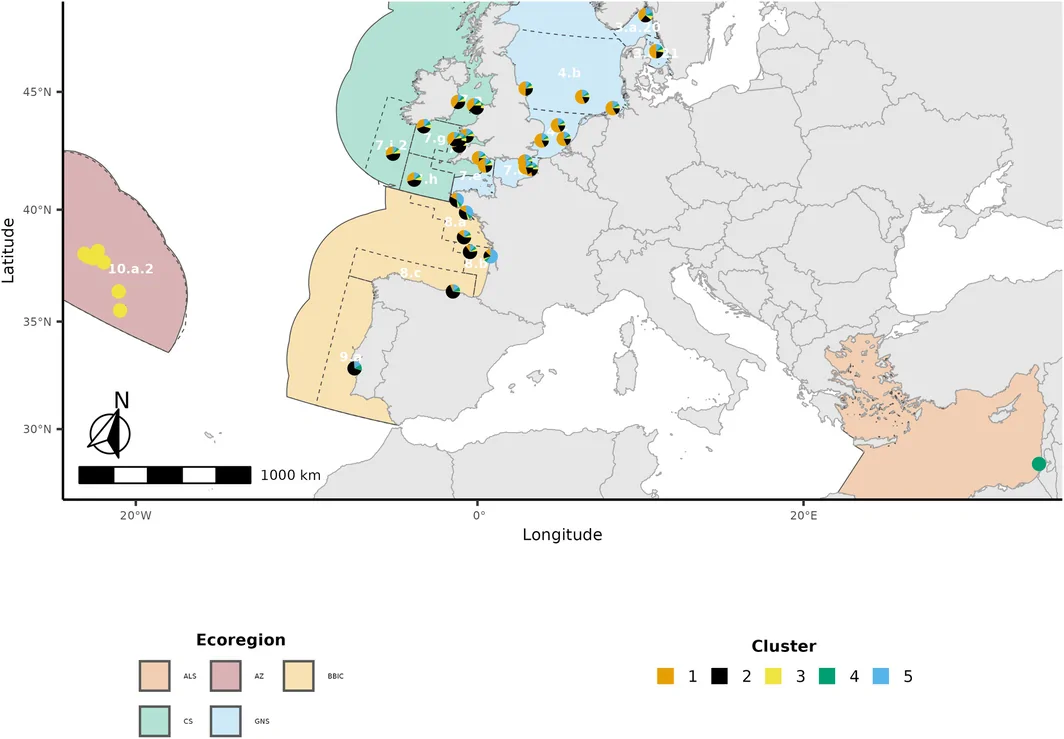
ADMIXTURE based Ancestry proportions of *Raja clavata* samples when classifying samples into ICES Division and coastal zone classifications for *K* = 5. Map delineates ecoregions (left legend). Colours in pie charts indicate cluster ancestry proportions (right legend). For abbreviations, see Table A1.

**FIGURE A17:**
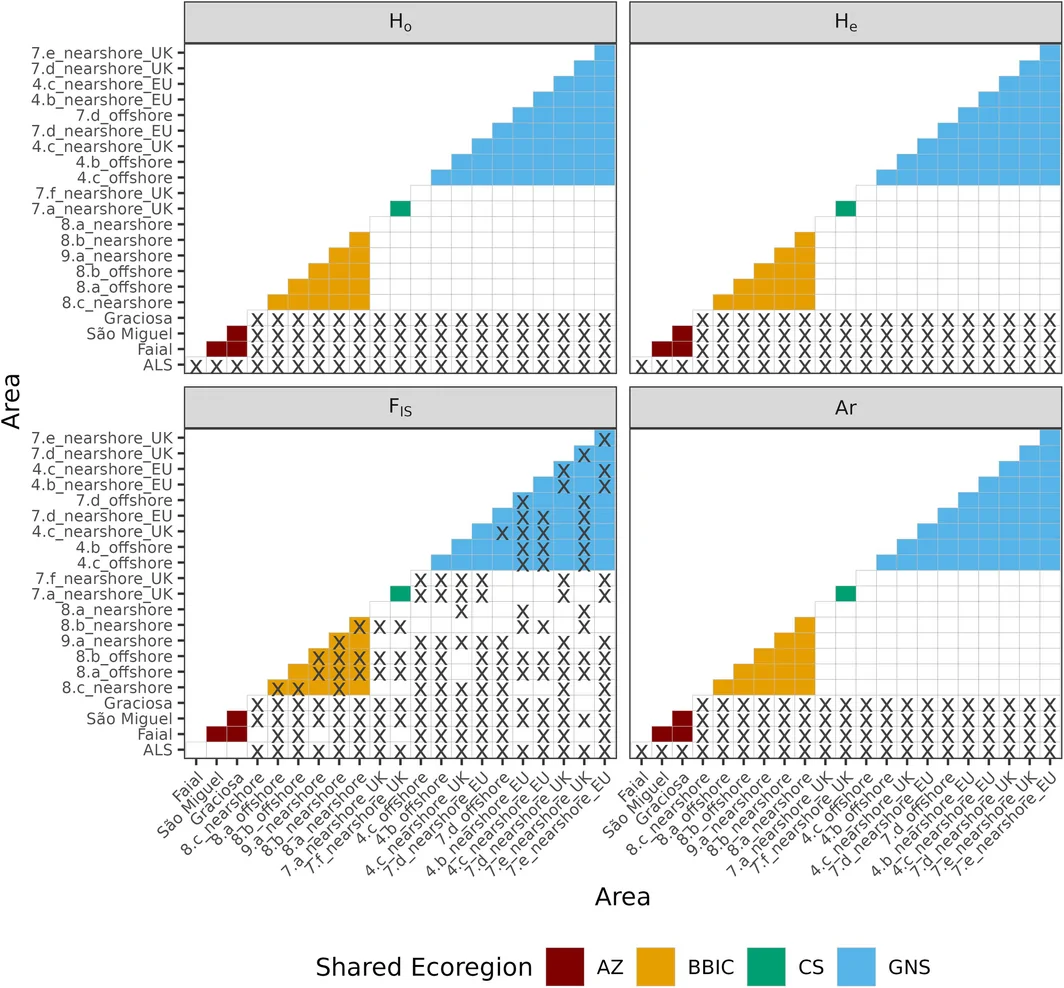
Dunn's test of genetic diversity estimates for *Raja clavata* across grouping based on ICES Division and coastal zone classification areas. Crosses (X) indicate significant *p*‐values (*p* < 0.05) after Holm's correction. For abbreviations, see Table A1.

**FIGURE A18:**
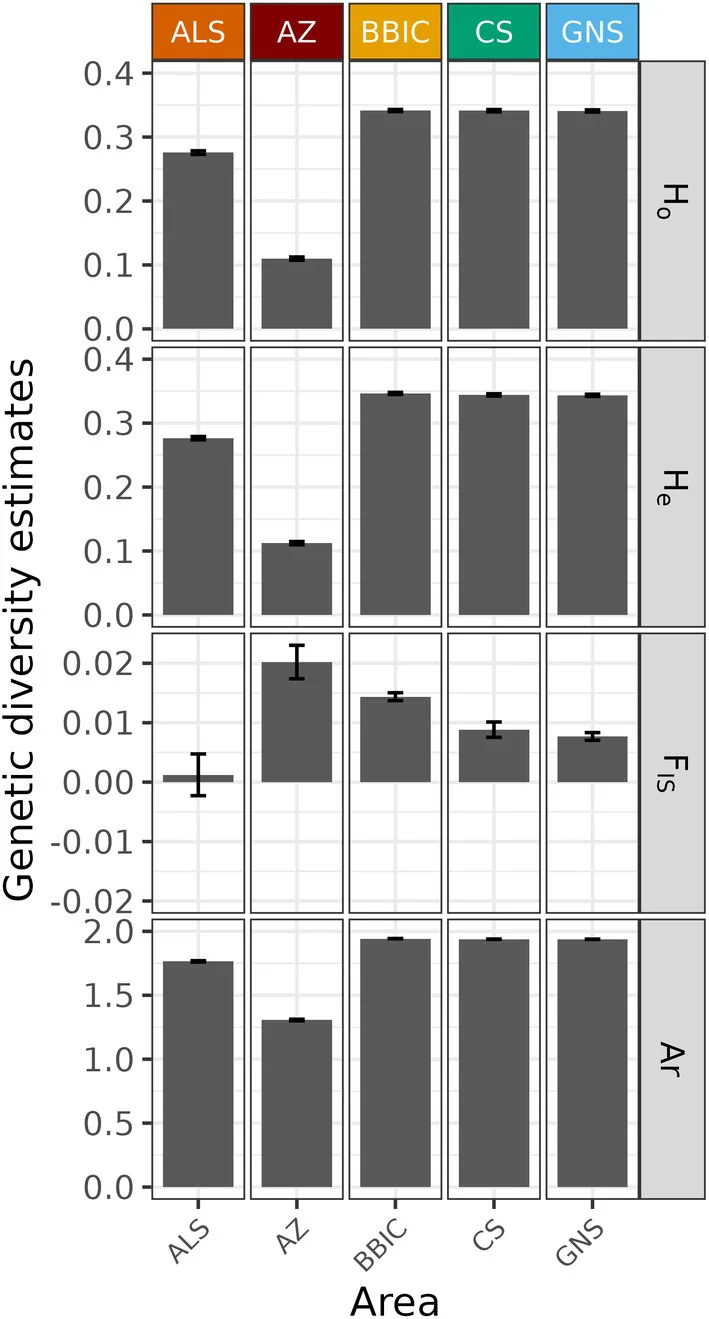
Genetic diversity estimates for *Raja clavata* samples grouped by ecoregions. Observed Heterozygosity (*H*_o_), expected heterozygosity (*H*_e_), Fixation index (*F*_IS_) and allelic richness (Ar) of populations grouped by ecoregions, with error bars indicating the standard error of the means. For abbreviations, see Table A1.

**FIGURE A19:**
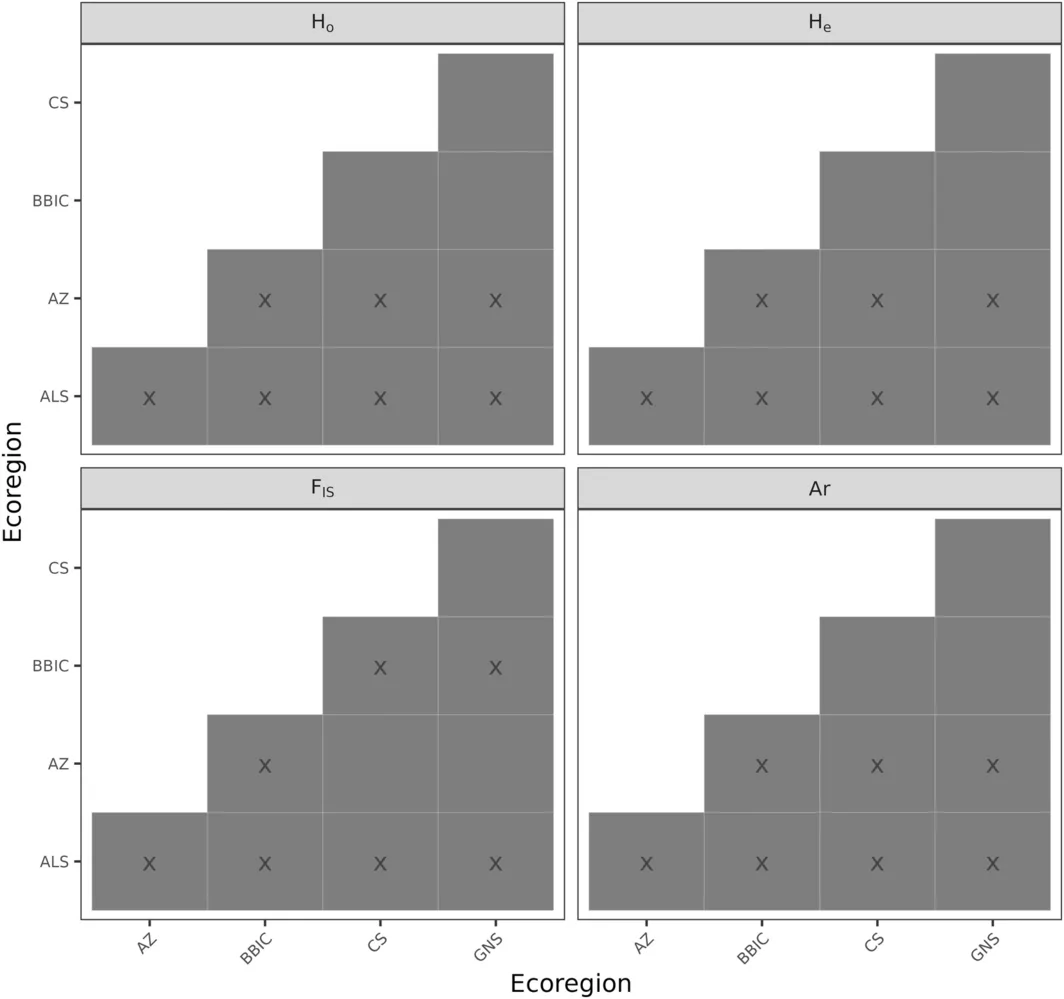
Dunn's test of genetic diversity estimates of *Raja clavata* across groupings based on ecoregion. Crosses (X) indicate significant *p*‐values (*p* < 0.05) after Holm's correction. For abbreviations, see Table A1.

**FIGURE A20:**
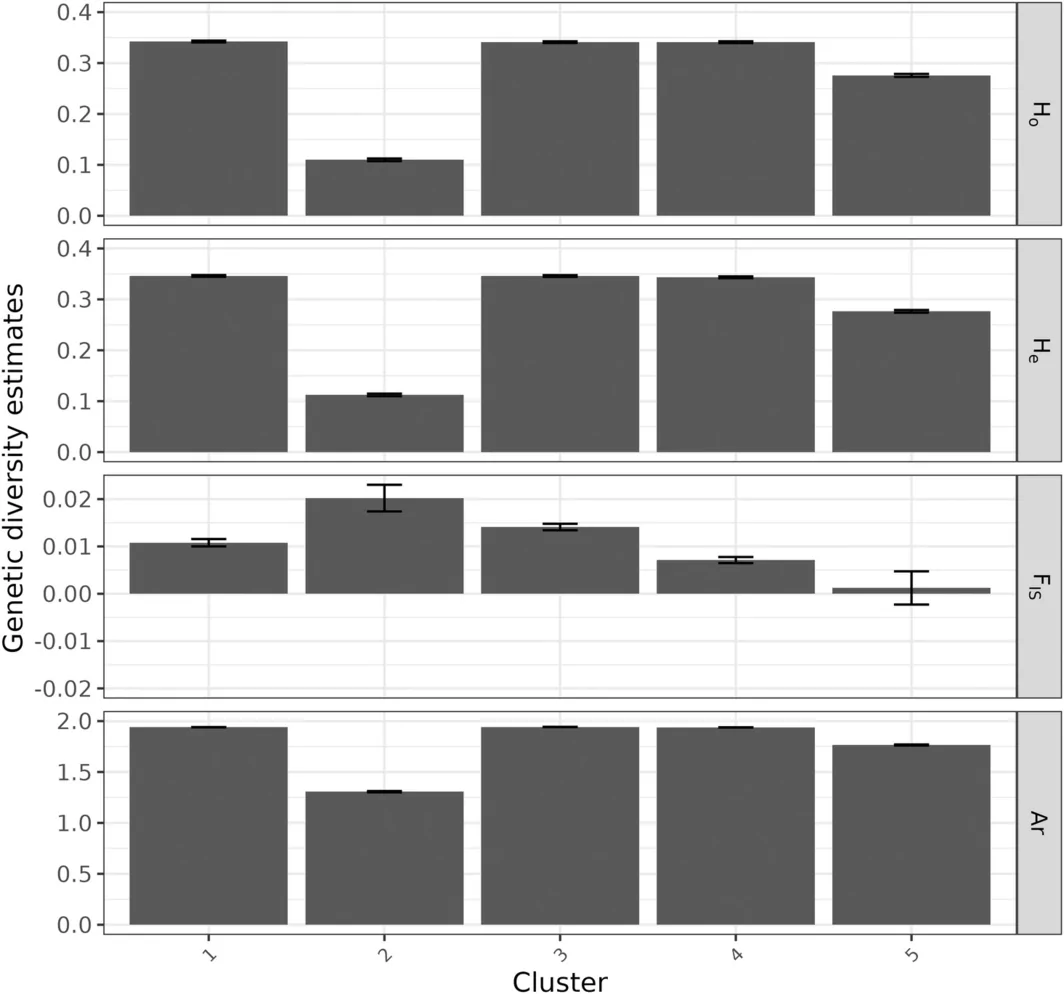
Genetic diversity estimates for *Raja clavata* , collected from the Northeast Atlantic and the Mediterranean Sea, across five suggested clusters (*K* = 5) from DAPC. Observed Heterozygosity (*H*_o_), expected heterozygosity (*H*_e_), Fixation index (*F*_IS_) and allelic richness (Ar), with error bars indicating the standard error of the means.

**FIGURE A21:**
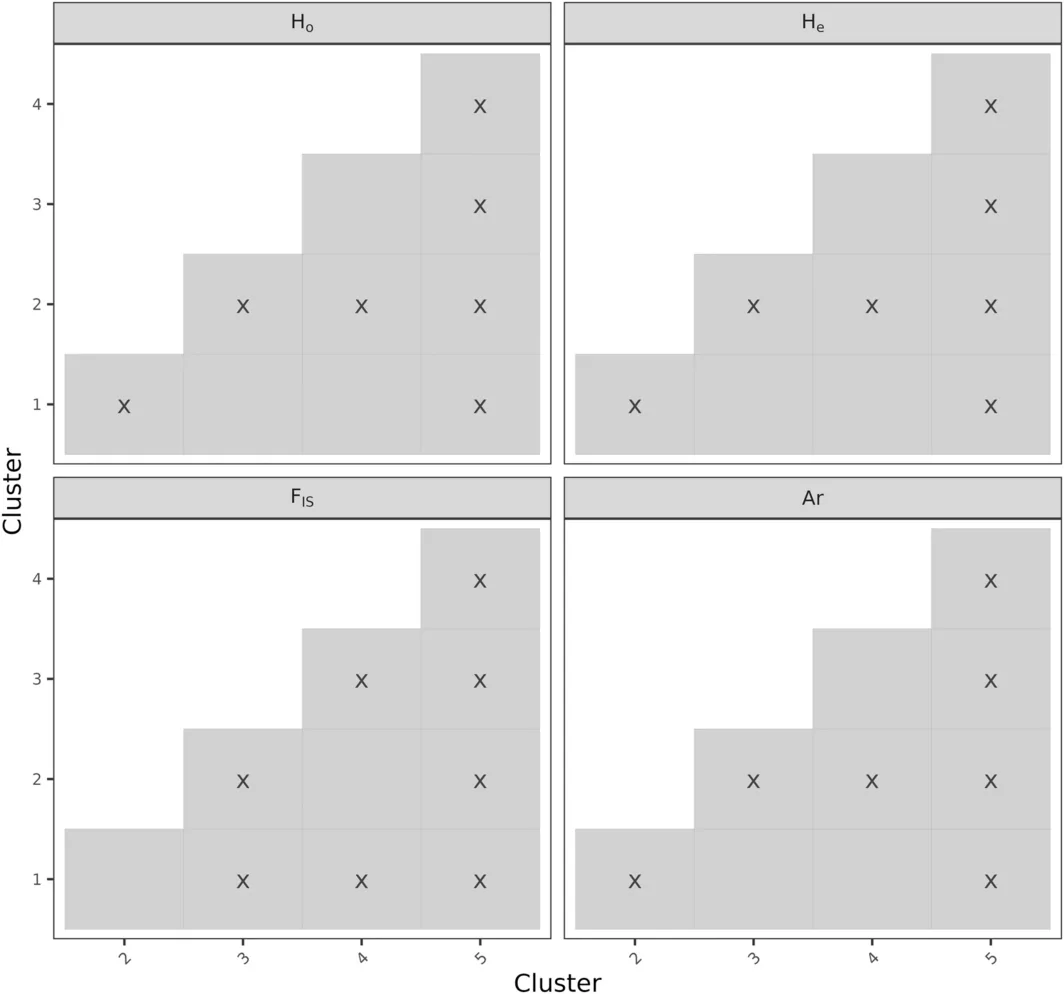
Dunn's test of genetic diversity estimates of *Raja clavata* , collected from the Northeast Atlantic and the Mediterranean Sea, across grouping based on five suggested clusters (*K* = 5) from DAPC. Crosses (X) indicate significant *p*‐values (*p* < 0.05) after Holm's correction.

## Data Availability

The data and code to reproduce the findings of this study are openly available in 4TU.ResearchData at http://doi.org/10.4121/fb54a10c‐494f‐4b88‐9413‐b741ad677962. The public data underlying this article were derived from sources in the public domain and consisted of depth data obtained from the European Marine Observation and Data Network (EMODnet) Bathymetry Portal: https://portal.emodnet‐bathymetry.eu.

## Appendix A

**TABLE A1:** Sex SNP identification results for genotyped samples of *Raja clavata* , collected from the Northeast Atlantic and Mediterranean Sea.

SNP	Heterozygosity excess (≥ 5×) in any sex	Sex marked SNPs on panel
RCL_P_1781	1	0
RCL_P_2373	1	0
RCL_P_2569	1	0
RCL_P_3244	1	0
RCL_P_3972	1	0
RCL_P_4615	1	0
RCL_P_4781	1	0
RCL_P_6491	1	0
RCL_P_8235_MAJ_T	1	0
RCL_P_8235_MIN_C	1	0
RCL_P_8298_MAJ_T	1	0
RCL_P_8298_MIN_C	1	0
RCL_P_9304_SexMAJ_T	1	1
RCL_P_9305_SexMAJ_A	1	1
RCL_P_9305_SexMIN_C	1	1
RCL_P_9308_Sex	1	1
RCL_P_9309_Sex	1	1
RCL_P_9310_Sex	1	1
RCL_P_9311_Sex	1	1
RCL_P_9312_Sex	1	1
RCL_P_9313_Sex	1	1
RCL_P_9317_Sex	1	1

*Note:* 0 indicates undetected and 1 indicates detected sex‐linked SNP. All SNPs suspected to be linked to sex were removed during the filtering steps.

**TABLE A2:** Overview of sample sizes of *Raja clavata* across ecoregion and ICES Division and coastal zone classification (Start), after removal of misidentified and intentional replicates and sample sizes used for genomic analyses after filtering.

Ecoregion	ICES Division and coastal zone classification	Sample period	Sample size		
			Start	Misidentified and intentional replicated samples removed	Post‐filtering
ALS	ALS	2022–2022	20	20	19
AZ	Faial	2001–2023	36	34	31
AZ	Graciosa	2001–2010	15	15	15
AZ	Pico	2010–2023	13	10	10
AZ	Santa Maria	2001–2011	4	4	1
AZ	São Jorge	2001–2011	14	14	14
AZ	São Miguel	2001–2011	20	20	20
AZ	Terceira	2001–2011	6	6	6
BBIC	8.a_nearshore	2011–2019	98	89	63
BBIC	8.a_offshore	2012–2018	1748	1662	1105
BBIC	8.b_nearshore	2013–2021	1031	951	906
BBIC	8.b_offshore	2012–2019	1113	1095	909
BBIC	8.c_nearshore	2014–2022	56	45	45
BBIC	9.a_nearshore	2015–2020	71	71	41
BBIC	9.a_offshore	2014–2014	1	1	0
CS	7.a_nearshore_IMN	2004–2004	1	1	0
CS	7.a_nearshore_IRL	2003–2004	24	24	11
CS	7.a_nearshore_UK	2003–2004	109	108	57
CS	7.a_offshore	2003–2004	15	15	11
CS	7.f_nearshore_UK	2003–2019	111	111	63
CS	7.f_offshore	2003–2003	1	1	1
CS	7.g_nearshore_IRL	2019–2019	1	1	1
CS	7.g_nearshore_UK	2003–2019	16	16	14
CS	7.g_offshore	2003–2019	11	11	10
CS	7.h_offshore	2017–2017	3	3	2
CS	7.j.2_offshore	2018–2019	7	7	7
GNS	3.a.20_offshore	2021–2021	2	2	2
GNS	3.a.21_nearshore_CE	2022–2023	17	13	13
GNS	4.b_nearshore_CE	2018–2018	48	48	45
GNS	4.b_nearshore_UK	2004–2020	2	2	2
GNS	4.b_offshore	2017–2023	489	479	393
GNS	4.c_nearshore_CE	2004–2023	103	103	83
GNS	4.c_nearshore_UK	2004–2023	473	472	327
GNS	4.c_offshore	2004–2023	2134	2111	1650
GNS	7.d_nearshore_CE	2004–2023	296	291	267
GNS	7.d_nearshore_UK	2004–2022	381	380	332
GNS	7.d_offshore	2004–2023	119	119	102
GNS	7.e_nearshore_CE	2015–2017	126	126	126
GNS	7.e_nearshore_UK	2003–2004	40	40	25
GNS	7.e_offshore	2003–2003	1	1	1
GNS	Unknown	2018–2023	368	329	172
Total		2001–2023	9144	8851	6902

Abbreviations: ALS = Aegean‐Levantine Sea, AZ = Azores, BBIC = Bay of Biscay and the Iberian Coast, CS = Celtic Seas, GNS = Greater North Sea.

**TABLE A3:** Dunn's test with Holm's correction of depths for DAPC without prior and grouping based on four clusters (*K* = 4), for genotyped samples of *Raja clavata* , collected across the Northeast Atlantic and Mediterranean Sea.

group1	group2	n1	n2	z‐score	*p*	*p*.adj
1	2	3409	97	−10.38	< 0.0001	< 0.0001
1	3	3409	1059	28.14	< 0.0001	< 0.0001
1	4	3409	2112	−52.78	< 0.0001	< 0.0001
2	3	97	1059	19.40	< 0.0001	< 0.0001
2	4	97	2112	−3.79	0.0002	0.0002
3	4	1059	2112	−65.10	< 0.0001	< 0.0001

**TABLE A4:** Dunn's test with Holm's correction of depths for DAPC without prior and grouping based on five clusters (*K* = 5), for genotyped samples of *Raja clavata* , collected across the Northeast Atlantic and Mediterranean Sea.

group1	group2	n1	n2	z‐score	*p*	*p*.adj
1	2	1058	97	−19.41	< 0.0001	< 0.0001
1	3	1058	2094	−65.11	< 0.0001	< 0.0001
1	4	1058	3409	−28.15	< 0.0001	< 0.0001
1	5	1058	19	−8.56	< 0.0001	< 0.0001
2	3	97	2094	−3.82	0.0001	0.0004
2	4	97	3409	10.38	< 0.0001	< 0.0001
2	5	97	19	0.31	0.7591	0.7591
3	4	2094	3409	52.77	< 0.0001	< 0.0001
3	5	2094	19	2.06	0.0399	0.0797
4	5	3409	19	−4.31	< 0.0001	< 0.0001

**TABLE A5:** sNMF based average cluster assignment probabilities of *Raja clavata* samples, collected across the Northeast Atlantic and Mediterranean Sea, obtained from LEA with 5 clusters (*K* = 5) by sub‐populations based on ICES Division and coastal zone classification.

ICES Division and coastal zone classification	Cluster 1	Cluster 2	Cluster 3	Cluster 4	Cluster 5
3.a.20_offshore	0.130	0.484	0.112	0.022	0.251
3.a.21_nearshore_CE	0.094	0.547	0.099	0.030	0.229
4.b_nearshore_CE	0.110	0.627	0.083	0.023	0.158
4.b_nearshore_UK	0.085	0.505	0.072	0.014	0.322
4.b_offshore	0.109	0.601	0.081	0.023	0.186
4.c_nearshore_CE	0.104	0.600	0.078	0.023	0.194
4.c_nearshore_UK	0.102	0.609	0.085	0.023	0.181
4.c_offshore	0.106	0.605	0.076	0.020	0.193
7.a_nearshore_IRL	0.096	0.438	0.072	0.031	0.363
7.a_nearshore_UK	0.113	0.422	0.056	0.024	0.386
7.a_offshore	0.093	0.427	0.065	0.034	0.380
7.d_nearshore_CE	0.111	0.574	0.076	0.024	0.215
7.d_nearshore_UK	0.113	0.576	0.074	0.021	0.217
7.d_offshore	0.102	0.581	0.077	0.022	0.218
7.e_nearshore_CE	0.353	0.165	0.106	0.022	0.354
7.e_nearshore_UK	0.112	0.536	0.067	0.031	0.254
7.e_offshore	0.126	0.608	0.024	0.006	0.235
7.f_nearshore_UK	0.120	0.434	0.066	0.025	0.355
7.f_offshore	0.062	0.366	0.032	0.030	0.511
7.g_nearshore_IRL	0.140	0.304	0.078	0.062	0.415
7.g_nearshore_UK	0.125	0.417	0.064	0.028	0.367
7.g_offshore	0.096	0.417	0.058	0.032	0.397
7.h_offshore	0.082	0.312	0.050	0.057	0.498
7.j.2_offshore	0.083	0.362	0.060	0.042	0.452
8.a_nearshore	0.333	0.161	0.109	0.018	0.380
8.a_offshore	0.136	0.169	0.071	0.022	0.603
8.b_nearshore	0.591	0.088	0.128	0.015	0.178
8.b_offshore	0.117	0.113	0.068	0.021	0.680
8.c_nearshore	0.162	0.047	0.123	0.003	0.666
9.a_nearshore	0.164	0.003	0.196	0.000	0.636
ALS	0.000	0.000	0.998	0.001	0.001
Faial	0.001	0.002	0.003	0.993	0.001
Graciosa	0.002	0.003	0.001	0.994	0.000
Pico	0.000	0.000	0.002	0.998	0.000
Santa Maria	0.000	0.000	0.000	1.000	0.000
São Jorge	0.001	0.003	0.001	0.995	0.000
São Miguel	0.004	0.006	0.002	0.986	0.001
Terceira	0.004	0.005	0.002	0.987	0.001

**TABLE A6:** sNMF based average cluster assignment probabilities of *Raja clavata* samples, collected from the Northeast Atlantic and Mediterranean Sea, obtained from LEA with 4 clusters (*K* = 4) by sub‐populations based on ICES Division and coastal zone classification.

ICES Division and coastal zone classification	Cluster 1	Cluster 2	Cluster 3	Cluster 4
3.a.20_offshore	0.126	0.543	0.030	0.301
3.a.21_nearshore_CE	0.106	0.633	0.034	0.227
4.b_nearshore_CE	0.142	0.705	0.024	0.129
4.b_nearshore_UK	0.080	0.623	0.012	0.284
4.b_offshore	0.136	0.684	0.025	0.155
4.c_nearshore_CE	0.132	0.688	0.025	0.155
4.c_nearshore_UK	0.131	0.687	0.025	0.156
4.c_offshore	0.133	0.689	0.023	0.156
7.a_nearshore_IRL	0.111	0.542	0.033	0.314
7.a_nearshore_UK	0.116	0.535	0.026	0.323
7.a_offshore	0.100	0.539	0.035	0.325
7.d_nearshore_CE	0.137	0.657	0.026	0.180
7.d_nearshore_UK	0.136	0.662	0.024	0.178
7.d_offshore	0.127	0.668	0.025	0.180
7.e_nearshore_CE	0.348	0.225	0.023	0.404
7.e_nearshore_UK	0.130	0.621	0.033	0.216
7.e_offshore	0.122	0.741	0.013	0.124
7.f_nearshore_UK	0.125	0.541	0.027	0.306
7.f_offshore	0.057	0.525	0.030	0.388
7.g_nearshore_IRL	0.155	0.394	0.070	0.381
7.g_nearshore_UK	0.139	0.522	0.030	0.309
7.g_offshore	0.099	0.531	0.035	0.335
7.h_offshore	0.081	0.431	0.057	0.430
7.j.2_offshore	0.080	0.488	0.044	0.388
8.a_nearshore	0.322	0.220	0.020	0.439
8.a_offshore	0.117	0.297	0.023	0.563
8.b_nearshore	0.590	0.088	0.016	0.306
8.b_offshore	0.092	0.251	0.022	0.635
8.c_nearshore	0.143	0.147	0.004	0.707
9.a_nearshore	0.155	0.023	0.001	0.821
ALS	0.000	0.000	0.000	1.000
Faial	0.001	0.003	0.996	0.001
Graciosa	0.001	0.002	0.996	0.000
Pico	0.000	0.000	0.999	0.001
Santa Maria	0.000	0.000	1.000	0.000
São Jorge	0.001	0.001	0.998	0.000
São Miguel	0.005	0.006	0.989	0.001
Terceira	0.001	0.007	0.990	0.002

**TABLE A7:** Mean cross‐validation error and mean loglikelihood of 10 replicates per cluster (*K*) from ADMIXTURE based admixture analyses.

*K*	Mean CV error	Mean log‐likelihood
1	0.575600	−34,335,793
2	0.565402	−33,990,604
3	0.563711	−33,922,090
4	0.563088	−33,887,918
**5**	**0.562937**	**−33,866,096**
6	0.563066	−33,852,417
7	0.563224	−33,839,450
8	0.563457	−33,827,471
9	0.563701	−33,815,865
10	0.563937	−33,804,441

*Note:* *K* with lowest cross‐validation error highlighted in bold.

**TABLE A8:** ADMIXTURE based average cluster assignment probabilities of *Raja clavata* samples, collected from the Northeast Atlantic and Mediterranean Sea, for five clusters (*K* = 5) by sub‐populations based on ICES Division and coastal zone classification.

ICES Division and coastal zone classification	Cluster 1	Cluster 2	Cluster 3	Cluster 4	Cluster 5
3.a.20_offshore	0.387	0.255	0.091	0.082	0.185
3.a.21_nearshore_CE	0.502	0.223	0.074	0.081	0.119
4.b_nearshore_CE	0.584	0.161	0.062	0.060	0.133
4.b_nearshore_UK	0.498	0.265	0.064	0.080	0.093
4.b_offshore	0.565	0.177	0.060	0.061	0.137
4.c_nearshore_CE	0.570	0.180	0.060	0.061	0.129
4.c_nearshore_UK	0.572	0.174	0.059	0.064	0.132
4.c_offshore	0.568	0.181	0.058	0.059	0.134
7.a_nearshore_IRL	0.397	0.365	0.075	0.069	0.094
7.a_nearshore_UK	0.373	0.363	0.070	0.065	0.129
7.a_offshore	0.375	0.373	0.080	0.068	0.104
7.d_nearshore_CE	0.533	0.210	0.062	0.060	0.135
7.d_nearshore_UK	0.543	0.207	0.059	0.057	0.134
7.d_offshore	0.544	0.201	0.059	0.062	0.134
7.e_nearshore_CE	0.172	0.345	0.047	0.070	0.366
7.e_nearshore_UK	0.497	0.251	0.068	0.055	0.130
7.e_offshore	0.527	0.231	0.067	0.064	0.111
7.f_nearshore_UK	0.400	0.339	0.066	0.063	0.133
7.f_offshore	0.289	0.460	0.085	0.052	0.114
7.g_nearshore_IRL	0.305	0.399	0.087	0.078	0.131
7.g_nearshore_UK	0.355	0.355	0.080	0.074	0.136
7.g_offshore	0.394	0.379	0.069	0.058	0.101
7.h_offshore	0.292	0.442	0.084	0.066	0.116
7.j.2_offshore	0.293	0.464	0.088	0.057	0.097
8.a_nearshore	0.168	0.372	0.041	0.073	0.345
8.a_offshore	0.147	0.596	0.058	0.068	0.131
8.b_nearshore	0.129	0.172	0.030	0.066	0.602
8.b_offshore	0.098	0.673	0.055	0.066	0.107
8.c_nearshore	0.066	0.667	0.022	0.096	0.149
9.a_nearshore	0.015	0.687	0.003	0.138	0.157
ALS	0.000	0.000	0.000	1.000	0.000
Faial	0.000	0.000	1.000	0.000	0.000
Graciosa	0.000	0.000	1.000	0.000	0.000
Pico	0.000	0.000	1.000	0.000	0.000
Santa Maria	0.000	0.000	1.000	0.000	0.000
São Jorge	0.000	0.000	1.000	0.000	0.000
São Miguel	0.000	0.000	1.000	0.000	0.000
Terceira	0.000	0.000	1.000	0.000	0.000
Unknown	0.570	0.183	0.059	0.060	0.128
